# Very long chain sphingolipids govern brain myelination by regulating oligodendrocyte differentiation and membrane microdomain integrity

**DOI:** 10.1186/s12967-026-07881-0

**Published:** 2026-02-19

**Authors:** Huan Sun, Luyue Mo, Mingjun Cao, Yanlin Tian, Lesong Mo, Zhen Ni, Shaohua Zhang, Xiahe Huang, Yingchun Wang, Sin Man Lam, Guanghou Shui

**Affiliations:** 1 Institute of Genetics and Developmental Biology, Chinese Academy of Sciences, Beijing, 100101 China; 2 University of Chinese Academy of Sciences, Beijing, 100049 China; 3 Jiangsu Key Laboratory of Molecular Targets and Intervention for Metabolic Diseases, LipidALL Technologies, Changzhou, 213022 China; 4 Guangzhou National Laboratory, Guangzhou, 510005 China; 5 Guangzhou Institute of Cancer Research, the Affiliated Cancer Hospital, Guangzhou Medical University, Guangzhou, 510095 China

**Keywords:** *CerS2*, VLC sphingolipids, Lipids, Myelin abnormalities, Oligodendrocytes

## Abstract

**Background:**

Myelin abnormalities, which have no effective treatment to-date, underlie numerous debilitating neurological disorders like Multiple Sclerosis (MS). While lipids constitute ~70% of myelin, the specific lipids critical for establishment of myelin sheath, and the mechanisms linking lipid metabolism to myelin assembly, have remained poorly defined, hindering therapeutic development.

**Methods:**

We generated central nervous system-specific (cKO-nestin) and oligodendrocyte-specific (cKO-OL) conditional knockout mice for *Ceramide Synthase 2* (*CerS2*), which synthesizes very long-chain (VLC; C22–C24) ceramides. A multi-omics approach combining lipidomics, proteomics, and high-resolution mass spectrometry imaging (MSI) was employed to delineate the lipid and protein aberrations upon *CerS2* knockout. Functional consequences were assessed using behavioral tests, electron microscopy, and in vitro myelination assays of dorsal root ganglion (DRG) neuron-oligodendrocyte precursor cells (OPCs) co-cultures.

**Results:**

*CerS2* knockout mice exhibited severe neurological phenotypes, including convulsions, premature lethality, and profound hypomyelination. Lipidomics revealed pathological lipid remodeling marked by drastic depletion of VLC sphingolipids and compensatory increases in shorter-chain (C16–C18) ceramides—a lipid profile clinically relevant to MS. MSI confirmed VLC sphingolipid loss was localized to disrupted white matter tracts. Crucially, wild-type brain lipid extracts, but not those from CerS2-knockout mice, rescued oligodendrocyte differentiation and myelination in vitro. Mechanistically, VLC sphingolipid loss ablated membrane microdomains, displacing myelin basic protein (MBP) from lipid rafts that jeopardizes normal myelination. Pharmacological disruption of microdomains recapitulated the myelination failure.

**Conclusion:**

Our findings define a novel pathogenic cascade where cell-autonomous loss of CerS2 impedes oligodendrocyte differentiation and depletes VLC sphingolipids, destabilizing membrane microdomains and impairing MBP localization, culminating in myelination failure. This work positions the CerS2-VLC sphingolipid axis as a potential therapeutic target for demyelinating diseases.

**Supplementary information:**

The online version contains supplementary material available at 10.1186/s12967-026-07881-0.

## Introduction

Myelin is a lipid-rich structure that ensheaths axons, which plays a fundamental role in the rapid and efficient relay of electrical signals within the brain. Beyond signal conduction, the myelin sheath also provides protection and nutrition to neurons [1, 2]. Myelination, the process of myelin generation and ensheathing around the axons of nerve cells, is executed by different cells. In the central nervous system (CNS), specialized glial cells known as oligodendrocytes myelinate multiple adjoining axons. Damage to myelin, in the form demyelination or myelination abnormalities, can detrimentally impair inter-neuronal communication, leading to neuronal dysfunction manifested in a range of complications such as neuropathies, convulsions, paralysis, and even death. As a prominent example of myelin disorder, multiple sclerosis (MS) is a chronic autoimmune, inflammatory, neurodegenerative disorder characterized by myelin damage affecting both the white and gray matter of the brain, with an estimated 2.8 million people being affected worldwide in 2020 [3]. The incidence of MS is twofold higher in women than men [3], with onset mostly at young adulthood (between 20 to 40 years old) [4]. Despite its prevalence, the precise etiology of MS has remained incompletely understood. In addition to myelin damage, inflammation constitutes a key pathological feature of MS [5]. Targeting inflammation has become a central therapeutic strategy against MS, despite being ineffective in close to 10% of these patients who exhibited little or no inflammation [6], underscoring a significantly unmet clinical need. There remains no effective cure for MS to-date, particularly therapies aimed at ameliorating demyelination, and existing medications only serve to slow down progression or manage symptoms but not reverse neurological damages [7]. Oligodendrocyte precursor cells (OPCs), which are present in the adult human nervous system and can differentiate into mature, myelinating oligodendrocytes [8], represent promising targets for regenerative therapies. Following demyelinating injury, OPCs serve as a cellular reservoir for generating new oligodendrocytes that underlie remyelination [8, 9]. Understanding the process of endogenous remyelination might be key to unlocking regenerative therapies against demyelinating diseases like MS.

Membrane microdomains, also known as lipid rafts, are cholesterol- and sphingolipid-enriched subdomains of the plasma membrane, typically ranging from 10 to 200 nm in size [10, 11]. Relative to neighboring membrane regions, these specialized microdomains are more highly ordered with increased thickness due to higher content of lipids containing long-chain fatty acids, and resistant to non-ionic detergents [10]. Membrane microdomains are involved in membrane fluidity, intracellular trafficking, signal transduction, and endocytosis [10, 12], as well as affect the sorting and interactions of membrane proteins [13]. Sphingolipids represent a class of fundamental membrane lipids, which includes ceramide (Cer), sphingomyelin (SM), sphingosine-1-phosphate (S1P), and various glycosphingolipids (GSLs) such as ganglioside (GM), sulfatide (SL), galactosylceramide (GalCer), glucosylceramide (GluCer) and lactosylceramide (LacCer). Dysregulation of sphingolipid metabolism is implicated in a variety of human diseases including cardiovascular diseases, diabetes, infectious diseases and cancers [14–17]. Sphingolipids are also crucial for normal brain development, and altered sphingolipid levels are associated with demyelinating diseases such as MS [18, 19]. For example, aberrant sphingolipid accumulation arising from defects in metabolic enzymes triggers demyelination [20]. Beyond structural roles as membrane constituents, sphingolipids are biologically active molecules that regulate key cellular events in the brain, such as nervous system development, myelination and myelin maintenance. Consequently, compositional changes in membrane sphingolipids can disrupt plasma membrane microdomains, potentially contributing to the pathogenesis of demyelinating disorders.

As the structural backbones for all complex sphingolipids, ceramides are functionally diverse and structurally comprise fatty acid linked to a sphingosine via amide bond [13]. In mammals, six isoforms of ceramide synthases (CerS) synthesize ceramides with distinct acyl chain lengths. Ceramide synthase 2 (CerS2) specifically synthesizes very long acyl chain (VLC) ceramides (C22–C24) [21]. The clinical significance of CerS2 is highlighted by a case of progressive myoclonic epilepsy in a patient with heterozygous *CerS2* deletion, leading to substantially reduced levels of VLC sphingolipids [22]. Investigating the function of CerS2 specific to the brain can unravel the role of VLC sphingolipids in myelination, and potentially uncover new treatment targets against demyelinating diseases [22]. Here we leveraged a multi-omics approach to dissect the role of *CerS2* knockout in myelination, supplemented with anatomical atlases that spatially map the global lipid remodeling resulting from CNS-specific *CerS2* deletion captured using high-resolution imaging mass spectrometry (IMS).

## Methods

### Animals

*CerS2* flox mice were generated according to our previous study [21]. Briefly, the flanking of the 2^nd^ and 9^th^ exon in *CerS2* gene was inserted in two loxp sites. The nestin-cre mice were gifted by Wei-Xiang Guo lab at the Institute of Genetics and Developmental Biology, Chinese Academy of Sciences [23]. The olig-cre mice were gifted by Zhi-Heng Xu lab at the Institute of Genetics and Developmental Biology, Chinese Academy of Sciences. The mice were raised in the Animal Center (SPF), Institute of Genetics and Developmental Biology (IGDB), Chinese Academy of Sciences, and all experiments were conducted in accordance with the requirements of the Experimental Animal Ethics Committee, Institute of Genetics and Developmental Biology, Chinese Academy of Sciences (Approval No. AP2024021).

### Behavioral experiment

Behavioral experiments were carried out in the behavioral center of the Animal Center as previously described [24–27]. Briefly, motor coordination and balance were evaluated using the rota-rod test. Depression-related behavioral despair was quantified by measuring the duration of immobility in the tail suspension test and the forced swimming test. The Y-maze experiment was used to test the spatial memory ability of mice. Social dominance hierarchy was assessed using the social dominance tube test, in which the ranking of cKO-nestin mice relative to co-feeding control mice was determined. All behavioral testing was performed during a consistent window within the animals’ active phase to control for the confounding effects of circadian rhythm on emotional state and despair-like behavior. Furthermore, considering the established influence of chronic social stress and gut-brain axis signaling on behavioral outcomes, environmental conditions and social housing were strictly standardized throughout the experimental period.

### Cell isolation and culture

Isolation of E14.5-E17.5 mice neural progenitor cell (NPC) cells and in vitro induction to OPC and oligodendrocytes as described in protocol [28, 29]. In brief, the cerebral cortex of all fetal mice were obtained. The extracted cerebral cortex was placed into 1 mL NPC medium (Dulbecco’s Modified Eagle Medium/Nutrient mixture F-12 (DMEM/F12) was added with 10 ng/mL epidermal growth factor (EGF), 10 ng/mL basic fibroblast growth factor (bFGF), 2% B27 supplement and 100 × penicillin-streptomycin (PS), and then gently broken down into homogenate. The homogenate was placed on ice for 2 min and filter with 40 μm filter to obtain single cell supernatant. The cells obtained from each mouse were cultured in 10 cm cell culture dishes. Half of the medium was replaced every 2 days, and NPC were grown into oligospheres. The oligospheres were dislodged into single cells using accutase. OPC cells were induced using OPC culture medium (DMEM/F12, 10 ng/mL platelet-derived growth factor-AA (PDGF-AA), 10 ng/mL bFGF, 2% B27 and 100 × PS), and OPC cells were obtained within 2 days. OPC induced oligodendrocytes after 6 days of OL medium (DMEM/F12, 2% B27 and 100 × PS), during which β-CD were added.

DRG neuron was isolated from P3-7 mice as described in protocol [30]. In brief, the DRG was isolated using tweezers under an anatomical microscope. They were placed in DRG medium (basic medium DMEM, 10% FBS and 100 × PS) and centrifuged for 5 min at 161 g and the supernatant was discarded. Next, 1 mL of Collagenase A/Dispase II was added and incubated in a 37 °C shaker for 20 min. After incubation, the mixture was centrifuged for 5 min at 161 g, and the supernatant was discarded. A total of 3 × 104 cells were seeded on 24-well plates pretreated with 1 mg/mL Laminin and cultured in DRG medium. After 2 days, the medium was changed into co-culture medium (DMEM/F12 was added with 10 ng/mL PDGF-AA, 10 ng/mL bFGF, 50 ng/mL nerve growth factor-beta (β-NGF), 2% stemPro and 100 × PS), then 3/4 of the medium was changed every 2 days, and 10 μM 5’-fluorodeoxyuridine (FUdR) was added into the culture medium to inhibit the growth of non-nerve cells.

The co-culture ratio of OPCs and DRG neurons that have been growing in vitro for 9 days is about 1:20 [31], during which β-CD were added. Staining analysis was performed after 1–2 weeks of co-culture. Data were analyzed using GraphPad Prism.

### Western blotting

Radioimmunoprecipitation assay (RIPA) lysate (including cocktail) was added to homogenize the tissue using a homogenizer. The homogenized tissue was sonicated in the ultrasonic instrument for 10 s with an interval of 10 s for 1 min. The sample was placed on ice for lysis for 30 min, centrifuged at 12,000 rpm for 15 min, and the supernatant was kept at 4 °C. Protein concentration was determined by bicinchoninic acid (BCA). Firstly, 5 × protein loading buffer was added to supernatant and boiled for 5 min prior to loading. The samples were loaded onto a gel and SDS-PAGE electrophoresis was performed. After SDS-PAGE electrophoresis, the samples on the gel were transferred onto a polyvinylidene difluoride (PVDF) membrane and western blotting was conducted using the following antibodies: CerS2 (Sigma, HPA027262), α-Tubulin (CST, 3873), MBP (Abcam, ab7349), Flotillin-1 (Abcam, ab41927). Protein imager was used to image the western blot and quantification of the protein expression levels were evaluated by gray value with Image J [21].

### Transmission electron microscopy and g-ratio analyses

The corpus callosum region of the brain from mice were dissected using mice brain matrix and immediately fixed with 2.5% glutaraldehyde overnight and post-fixed with 1% osmium tetroxide for 40 min at 4 °C. Samples were then stained with 3% uranyl acetate for 30 min at room temperature, washed with deionized water five times for 10 min each round, and dehydrated in a series of acetone treatments and infiltrated in embed-812 resin. The embedded tissues were cut into 70 nm slices and observed using a transmission electron microscope (TEM) (JEM 1400) at 80 kV. The number of myelin sheaths in individual TEM sections was counted, and g-ratios were calculated as the diameter of the axon divided by the diameter of the axon and its surrounding myelin sheath using ImageJ [32].

### Immunofluorescence

Mice brain sections or cells were fixed in 4% paraformaldehyde (PFA). The samples were blocked in 5% goat serum with 0.5% Triton-100 in phosphate-buffered saline (PBS) for 1 h at room temperature and incubated overnight at 4 °C with the MBP antibody (1:200, Biolegend: 836504), Neurofilament 200 (1:800, Sigma: N4142). Brain sections or cell wells were washed three times with PBS for 5 min and incubated with the secondary antibodies (Alexa 488 conjugated or Alexa-568 conjugated, 1:1000, Thermo Fisher Scientific) at room temperature for 1 h. Images were acquired using Zeiss Observer Z1 inverter microscope.

### Brain tissue sectioning

Frozen mouse brain tissues were securely fixed on the cryostat cutting stage. All tissue sections were cut at a thickness of 10 µm using a Lecia CM1950 cryostat (Leica Microsystems GmBH, Wetzlar, Germany) at −20 °C and subsequently mounted onto standard glass slides (CITOTEST). The slides were then placed in a vacuum desiccator and dried for 1 hour.

### Microdomains isolation

Membrane microdomains separation mainly refers to experimental methods [33, 34]. Isolated brain tissue was ground in the grinder until it was homogenized. Homogenate samples were centrifuged at 1000 g for 10 min at 4 °C. The supernatant was centrifuged in an ultracentrifuge at 200,000 g for 18 h at 4 °C using sucrose density gradients (5%, 35%, and 42.5%) membrane fractionation. The density gradient stratification was photographed and each layer was carefully collected for subsequent identification.

### Omics analyses

Lipidomics analyses were performed as described [32] and data were expressed in μmol/g mice brain weight. The phospholipid and sphingolipid omics were conducted on the AB SCIEX JasperTM HPLC-Triple Quad 4500MD system, and the triglyceride omics was analyzed on the Agilent 1260-AB SCIEX QTrap 5500 liquid mass spectrometer. Proteomics analysis was performed using Orbitrap Fusion Lumos Tribrid Mass Spectrometer (Thermo Scientific Rockford IL Waltham MA) coupled to Easy-nLC 1000 in data-dependent mode. Protein groups KEGG pathway analyzed on Webgestalt [35]. The other protein groups analyzed as described [36].

### DESI-MSI analysis

The DESI-MSI experiments were performed using a commercial DESI XS ion source (Waters), a high-resolution Q-TOF mass spectrometer (SELECT SERIES Cyclic IMS, Waters) coupled to a microflow solvent delivery system (ACQUITY UPLC M-Class μ BSM, Waters) [37]. In brief, imaging was carried out in positive ion mode with the following parameters: spatial resolution set at 10 μm step size, solvent flow rate at 250 nL·min^− 1^ and scan speed of 40 μm/s. The samples were separated on a nanoEase BEH C18 Column. For mass calibration, leucine enkephalin (LE) was added as an internal lock mass calibrant. Raw MSI data were acquired using HDI software (v1.8, Waters), and 2000 ions were extracted for image generation. Elemental compositions were assigned via MassLynx (v4.2, Waters).

### MALDI-MSI analysis

The MALDI-MSI experiments were performed using the iMScope QT instrument (Shimadzu, Kyoto, Japan) with an atmospheric pressure matrix-assisted laser desorption/ionization (AP-MALDI) source on LCMS-9050 Q-TOF mass spectrometer. The target region was located using the integrated optical microscope (iMScope QT), followed by Nd:YAG laser irradiation (355 nm).The laser beam diameter was set to 2 instrument units (~25 μm) with a fixed intensity of 68. Scanning proceeded at 30 μm pitch, with each pixel receiving 100 laser pulses (5,000 Hz repetition rate). Mass spectra were acquired in the range m/z 500–1000 at a detector voltage of 2.34 kV. Data processing and mass image reconstruction were conducted with IMAGEREVEALTM MS (Shimadzu).

### qRT-PCR

Total RNAs were extracted from brain using total RNA extraction kit (TIANGEN, Cat No: DP419) and cDNA was synthesized using the iScript cDNA Synthesis Kit (Bio-Rad, Cat No 1725120). The quantitative reverse transcription polymerase chain reaction (qRT-PCR) was performed using the SYBR Green PCR kit (Bio-Rad, Cat No 1708891) on a Bio-Rad CFX Connect 384-Real-Time PCR Detection machine. Relative expression values of genes were normalized to those of β-actin [32]. Gene-specific primers used for amplification were listed in Supplementary Table 1 .

## Results

### CNS-specific deletion of *CerS2* in mice recapitulates key features of demyelinating disease presented with neurological deficits

**Fig. 1:**
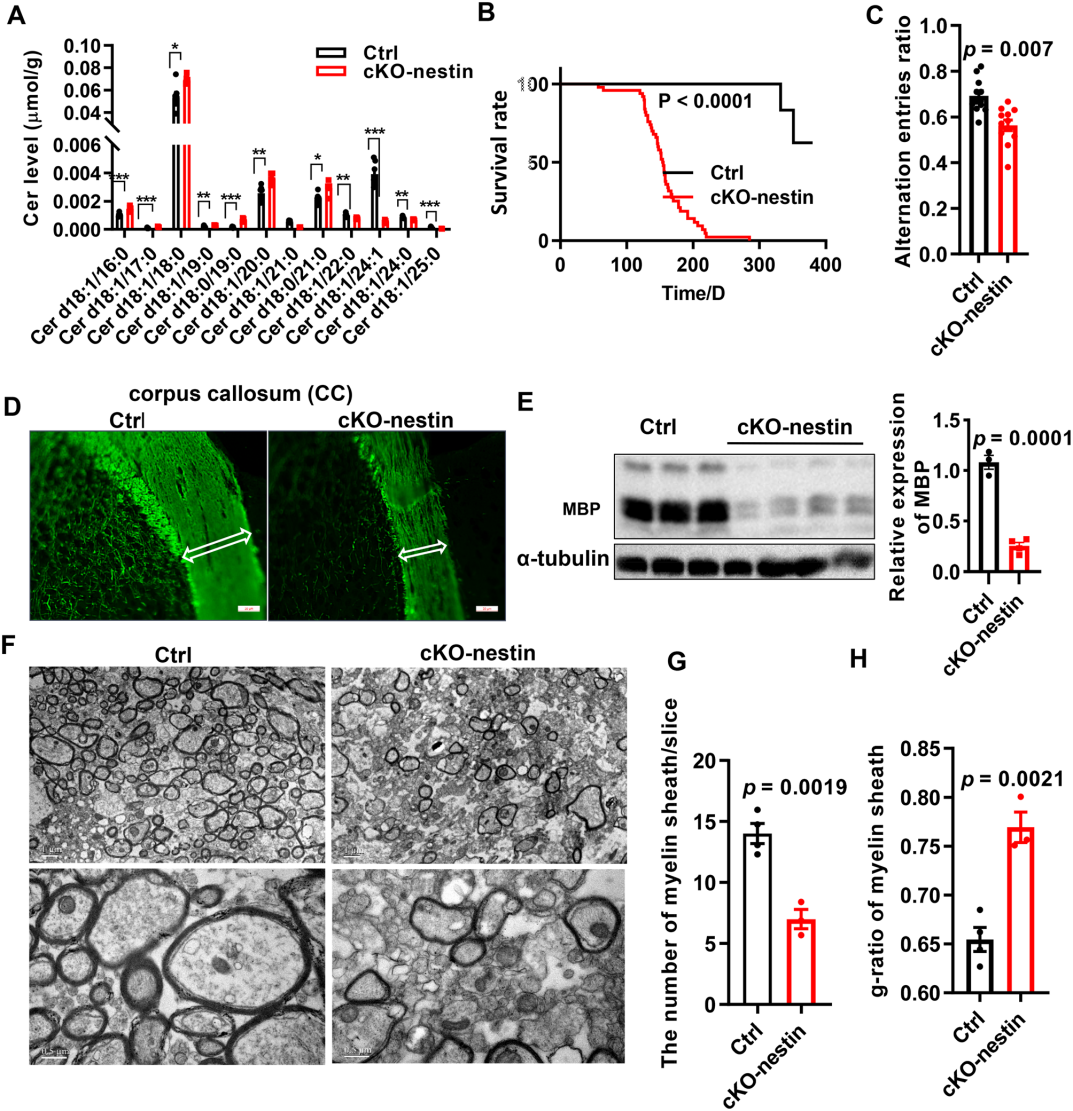
; pathological changes and myelination abnormalities in cKO-nestin mice. (**A**) Level of ceramides with different chain lengths in the brain. Lipidomics samples were extracted from 3-month-old male mice, *n* = 7 for Ctrl, *n* = 5 for cKO-nestin mice. (**B**) Statistical analysis of survival curves of cKO-nestin mice and control, *n* = 19 for male Ctrl and cKO-nestin mice, *n* = 28 for female Ctrl and cKO-nestin mice. (**C**) Proportion of spontaneous arm alternating of mice in Y maze test (number of arms alternating/(total number of arms entering − 2)), *n* = 11 for Ctrl, *n* = 12 for cKO-nestin mice. (**D**) Images of MBP immunofluorescence staining in brain sections of 3-month-old male mice, *n* = 3 for Ctrl and cKO-nestin mice. The thickness of corpus callosum are indicated with white double open arrows, scale, 20 μm. (**E**) Western blot of mice corpus callosum. MBP (upper) and α-tubulin (lower), right is the barplot illustrating the expression of MBP, *n* = 3 for Ctrl, *n* = 4 for cKO-nestin mice. (**F**) EM of corpus callosum in cKO-nestin mice and Ctrl, scales bar, 1 μm or 0.5 μm, respectively. (**G**) The number of myelin sheath in cKO-nestin mice and Ctrl, *n* = 4 for Ctrl, *n* = 3 for cKO-nestin mice. (**H**) G-ratio results of cKO-nestin mice and Ctrl, *n* = 4 for Ctrl, *n* = 3 for cKO-nestin mice, and for each mouse 10–20 EM images were analyzed. Significance was calculated using two-tailed *t* test, * *p* < 0.05; ** *p* < 0.01; *** *p* < 0.001. cKO-nestin, nervous system-specific *CerS2* knockout; Cer, ceramide; MBP, myelin basic protein; EM, electron microscopy; cKO, conditional knockout

*CerS2* null mice exhibit abnormal myelination, convulsions and lethality [38, 39]. To explore the function of *CerS2* within the CNS, we generated CNS-specific conditional knockout mice (*CerS2*^Flox/Flox^; Nestin Cre, hereafter as cKO-nestin) and used their littermates as controls (Ctrl) (Supplementary Fig. 1A). As expected, we observed significant reductions in the brain levels of CerS2 and its characteristic products – VLC ceramides (C22–C24), accompanied by compensatory increases in C16 and C18 ceramides (Fig. 1A and Supplementary Fig. 1B). The cKO-nestin mice exhibited significantly lower body weight than controls (Supplementary Fig. 1C). Commencing from approximately 4 months of age, cKO-nestin mice developed convulsions (Supplementary Video 1), with visible damages in the corpus callosum (CC) (Supplementary Fig. 1D). Mortality increased sharply from around 5 months of age (Fig. 1B). The onset of convulsion and death occurred earlier in female mice, although the difference between sexes did not reach statistical significance (Fig. 1B).

To characterize psychomotor functional consequences of *CerS2* deletion, we subject the cKO-nestin mice to a battery of behavioral tests. cKO-nestin mice exhibited significant spatial memory impairment in the Y-maze test (Fig. 1C), with no apparent irregularities in locomotion, general anxiety, depressive-like symptoms or social dominance based on the rotarod, open field, forced swim and tube tests (Supplementary Fig. 1E-I). To specifically assess social hierarchy, we performed the tube test in two distinct settings: first, by housing one cKO mouse with three Ctrl mice to evaluate competitive rank in a mixed-genotype group (Supplementary Fig. 1 H); and second, by housing four cKO mice together to determine their intrinsic ability to establish a social hierarchy among themselves (Supplementary Fig. 1I). In both paradigms, cKO mice did not show statistically significant deficits in social dominance compared to controls.Consistent with findings based on lipidomics and behavioral assays, neuropathological analyses revealed profound hypomyelination in cKO-nestin mice. Immunofluorescence staining for myelin basic protein (MBP) showed a thinner CC and shorter myelinated axonal lengths in the cerebral cortex (Fig. 1D). These were supported by appreciable reductions in MBP protein levels in the brain of cKO-nestin mice (Fig. 1E).

We next performed electron microscopy (EM) for ultrastructural examination of myelin sheaths. In the CC of cKO-nestin mice, the number of myelin sheaths per unit area was markedly reduced. Furthermore, existing sheaths were noticeably thinner compared to control mice (Fig. 1F–H).

### *CerS2* deficiency in oligodendrocytes drives progressive myelin abnormalities

**Fig. 2:**
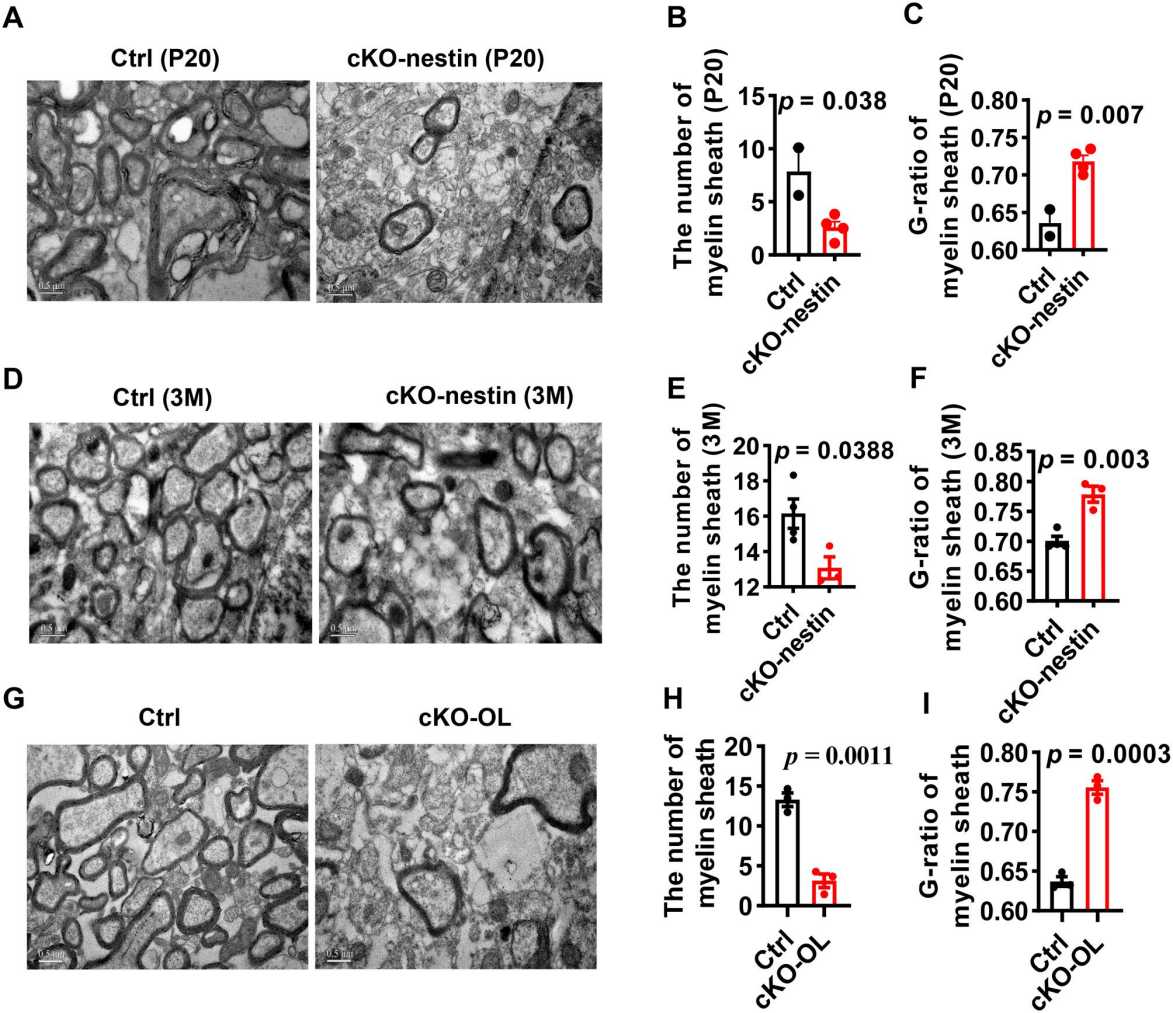
EM examination of myelination abnormalities in cKO-nestin and cKO-OL mice. (**A**) EM images of P20 mice corpus callosum. (**B**) The number of myelin sheath in P20 mice corpus callosum. (**C**) The g-ratio of myelin sheath in P20 mice corpus callosum, *n* = 4 for Ctrl, *n* = 2 for cKO-nestin mice. (**D**) EM images of 3-month-old mice corpus callosum EM. (**E**) The number of myelin sheath in 3-month-old mice corpus callosum. (**F**) The g-ratio of myelin sheath in 3-month-old mice corpus callosum, *n* = 4 for Ctrl, *n* = 3 for cKO-nestin mice. (**G**) EM images of cKO-OL mice and Ctrl corpus callosum, (**H**) The number of myelin sheath in cKO-OL mice and Ctrl mice corpus callosum, (**I**) The g-ratio of myelin sheath in cKO-OL mice and Ctrl mice corpus callosum, *n* = 3 for Ctrl and cKO-nestin mice. Scale bar, 0.5 μm. Significance was calculated using two-tailed *t* test. EM, electron microscopy; P20, postnatal day 20; cKO-nestin, nervous system-specific *CerS2* knockout; cKO-OL, oligodendrocyte specific *CerS2* knockout

The late onset of convulsions (~4 months) in cKO-nestin mice contrasted with the early initiation of myelination (postnatal day 15) in mice. We then sought to determine when myelin defects first emerge by performing EM ultrastructural analyses on the brain sections from postnatal day 20 (P20) and 3-month old cKO-nestin mice. EM images revealed significant reductions in both the number and thickness (g-ratio) of myelin sheaths in cKO-nestin mice as early as P20. The myelin deficit persisted and worsened in 3-month-old mice (Fig. 2A–F). Consistent with these findings, fast blue staining confirmed a marked decrease in myelin intensity and thickness in 3-month-old cKO-nestin mice (Supplementary Fig. 2A-C). These results showed that hypomyelination in CerS2 knockout mice is an early, primary defect that deteriorates over time, instead of secondary consequence from later-onset convulsions. Given that oligodendrocytes represent the predominant constituent cells of myelin sheath surrounding axons within the CNS, we generated oligodendrocyte-specific knockout of *CerS2* (cKO-OL) to establish a causal link between oligodendrocytes CerS2 function and the observed myelin pathology. The cKO-OL mice recapitulated the key phenotypes of the broader CNS knockout, which included reduced body weight, convulsions, increased lethality and impaired myelin formation (Fig. 2G–I and Supplementary Fig. 2D-E). The recapitulation of the key phenotypes—including hypomyelination, convulsions, and increased lethality—in the oligodendrocyte-lineage-restricted cKO-OLmice demonstrates that CerS2 deficiency within oligodendrocytes is sufficient to drive the pathology. While the broader CNS-specific deletion (cKO-nestin) could, in principle, involve secondary effects from other neural cell types, the convergence of the core myelin and neurological phenotypes across both models strongly argues that the loss of VLC sphingolipids primarily exerts a cell-autonomous effect within oligodendrocytes, directly impairing their myelination capacity. Therefore, the abnormal myelination is ascribed to oligodendrocyte-autonomous CerS2 deficiency, which directs our subsequent investigation toward oligodendrocyte-intrinsic mechanisms.

### *CerS2* deficiency dramatically disrupts the brain sphingolipid landscape

**Fig. 3:**
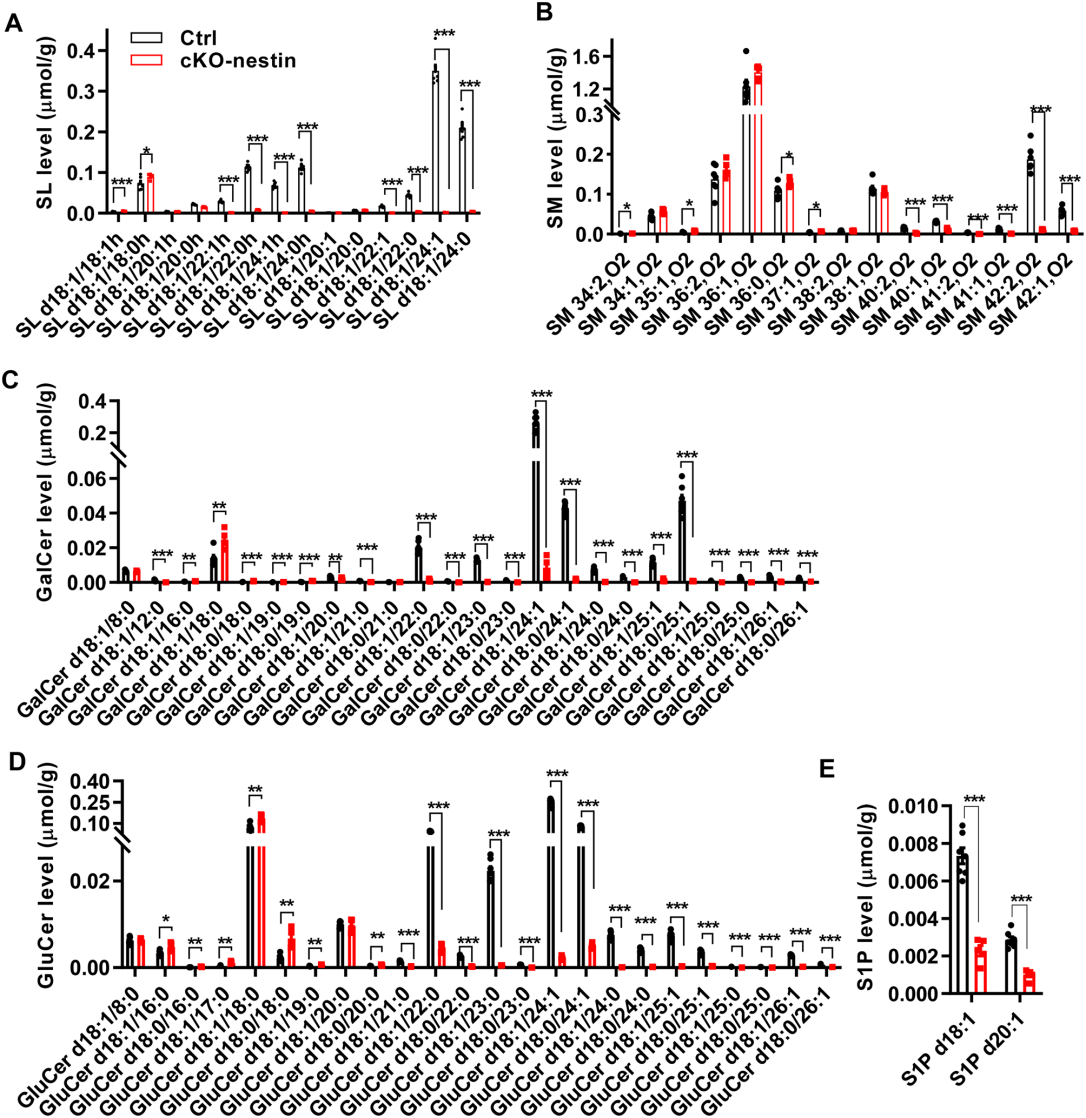
Lipid aberrations in cKO-nestin mice. Changes in the lipid composition of (**A**) SL, (**B**) SM, (**C**) GalCer, (**D**) GluCer and (**E**) S1P in cKO-nestin mice brain. Lipid samples were extracted from 3-month-old male mice, *n* = 7 for Ctrl, *n* = 5 for cKO-nestin mice and significance was calculated using two-tailed *t* test, * *p* < 0.05; ** *p* < 0.01; *** *p* < 0.001. SL, sulfatide; SM, sphingomyelin; GalCer, galactosylceramide; GluCer, glucosylceramide; S1P, sphingosine-1-phosphate; cKO-nestin, nervous system-specific *CerS2* knockout

Given that CerS2 is the primary synthase for VLC ceramides, we comprehensively analysed the brain lipidomes of cKO-nestin mice. As expected, VLC ceramides (C22–C24), the corresponding SM species and downstream complex glycosphingolipids, including SL, GluCer and GalCer possessing C22–C24 fatty acyls were significantly decreased. These were accompanied by compensatory increases in sphingolipids with shorter C16–C18 acyl chains (Fig. 3A–D). In addition, levels of sphingosine-1-phosphate (S1P) species were significantly reduced in cKO-nestin mice (Fig. 3E). Beyond the sphingolipidome, stark reductions across all individual phosphatidic acid (PA) species were observed for cKO-nestin mice, while saturated free fatty acids (FFA) C16:0 and C18:0 were significantly elevated (Supplementary Fig. 3A-B and Supplementary Table 2). Notably, the substantial remodeling of brain lipidomes occurred without alterations in the mRNA levels of key relevant biosynthetic enzymes, such as UGCG, B4GALT6 (Supplementary Fig. 3D). Taken together, these findings suggest that the massive lipid remodeling observed in cKO-nestin mice primarily resulted from reductions in VLC ceramides, which serve as a critical hub to maintain the overall brain lipid architecture essential for myelination.

### Spatial lipidomics reveals region-specific deficiency of VLC sphingolipids in cKO-nestin mice

**Fig. 4:**
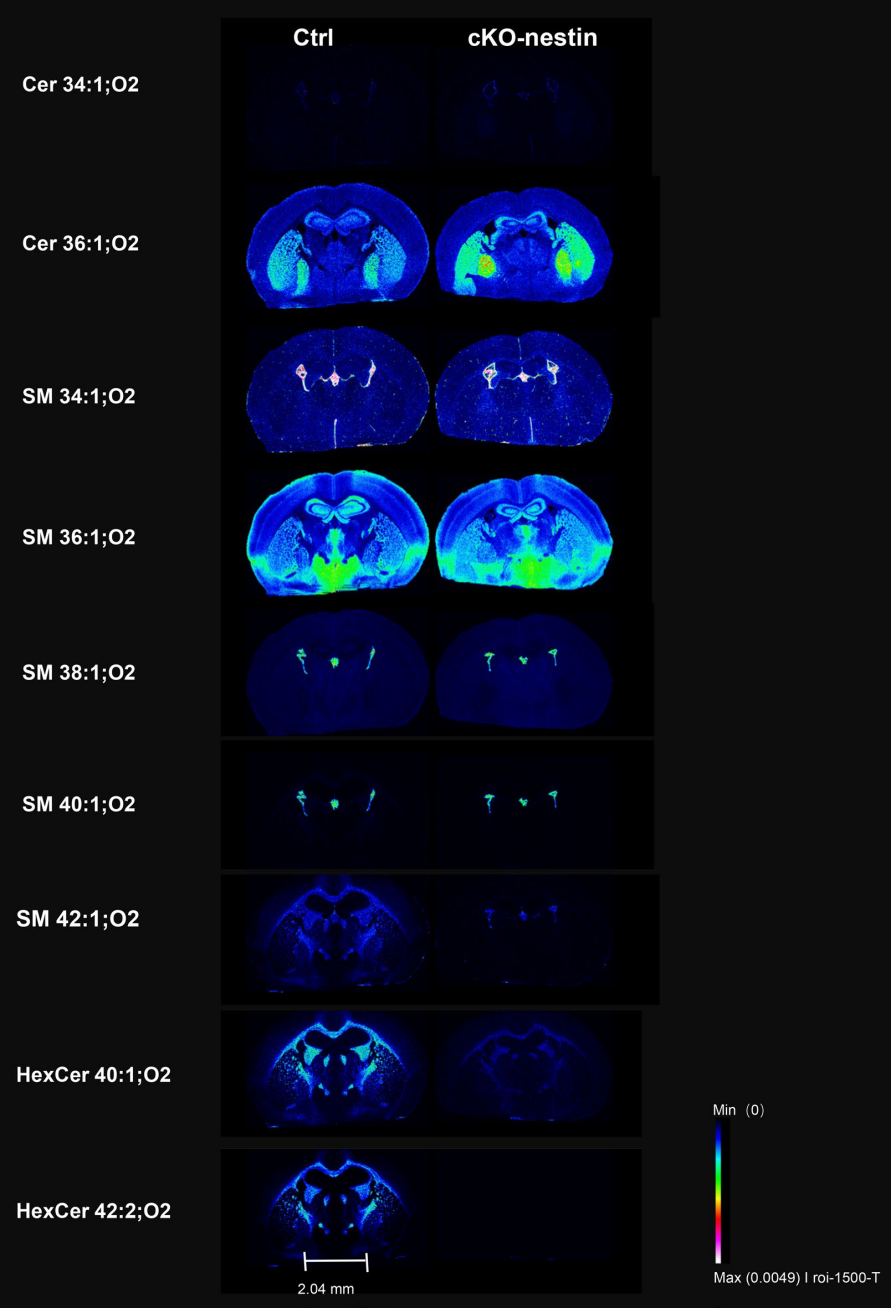
DESI-MSI of spatial lipid distribution across brain regions between Ctrl and cKO-nestin mice. Frozen mice brain tissues were cryosectioned at 10 µm thickness, and mass spectrometry images were acquired using a Waters mass spectrometer. The intensity of colors denotes the magnitude of lipid abundances. Scale bar: 2.04 mm. Data acquisition was performed using the m/z values of protonated parent ions as shown, unless otherwise specified. DESI-MSI, desorption electrospray ionization mass spectrometry imaging; cKO-nestin, nervous system-specific *CerS2* knockout; Cer, ceramide; SM, sphingomyelin; HexCer, hexosylceramide

To map the anatomical distribution of global lipid remodeling quantitated in the brain of cKO-nestin mice, we performed desorption electrospray ionization coupled mass spectrometry imaging (DESI-MSI) on a SELECT SERIES Cyclic IMS from Water Corporation, which achieved spatial resolution of 10 µm (Fig. 4). Our high-resolution IMS confirmed marked reductions of key VLC sphingolipids – including SM 42:1;O2, HexCer 40:1;O2 and HexCer 42:2;O2, in the brain of cKO-nestin mice. These deficiencies were localized precisely to major white matter tracts, including the corpus callosum (a critical region for myelination), as well as external and internal capsules of the brain. Other myelinated regions, such as the fimbria of the hippocampus and stria medullaris, also exhibited pronounced drop in VLC sphingolipid intensities (Fig. 4). Importantly, spatial patterns of VLC sphingolipid changes were replicated in independent experiments using matrix-assisted laser desorption/ionization-MSI (MALDI-MSI) (Supplementary Fig.4). Conversely, sphingolipids with shorter acyl chains (C16-18), such as Cer 36:1;O2, SM 34:1; O2 and SM 36:1;O2, were increased in the striatum, globus pallidus and hippocampus (Fig. 4 and Supplementary Fig.4). MSI findings henceforth establish a direct spatial colocalization between deficiencies of CerS2-derived VLC sphingolipids and the sites of myelin disruption, underscoring the role of CerS2-dependent lipid metabolism in maintaining overall myelination.

### VLC ceramides deficiency disrupts membrane microdomains and displaces myelin basic protein

**Fig. 5:**
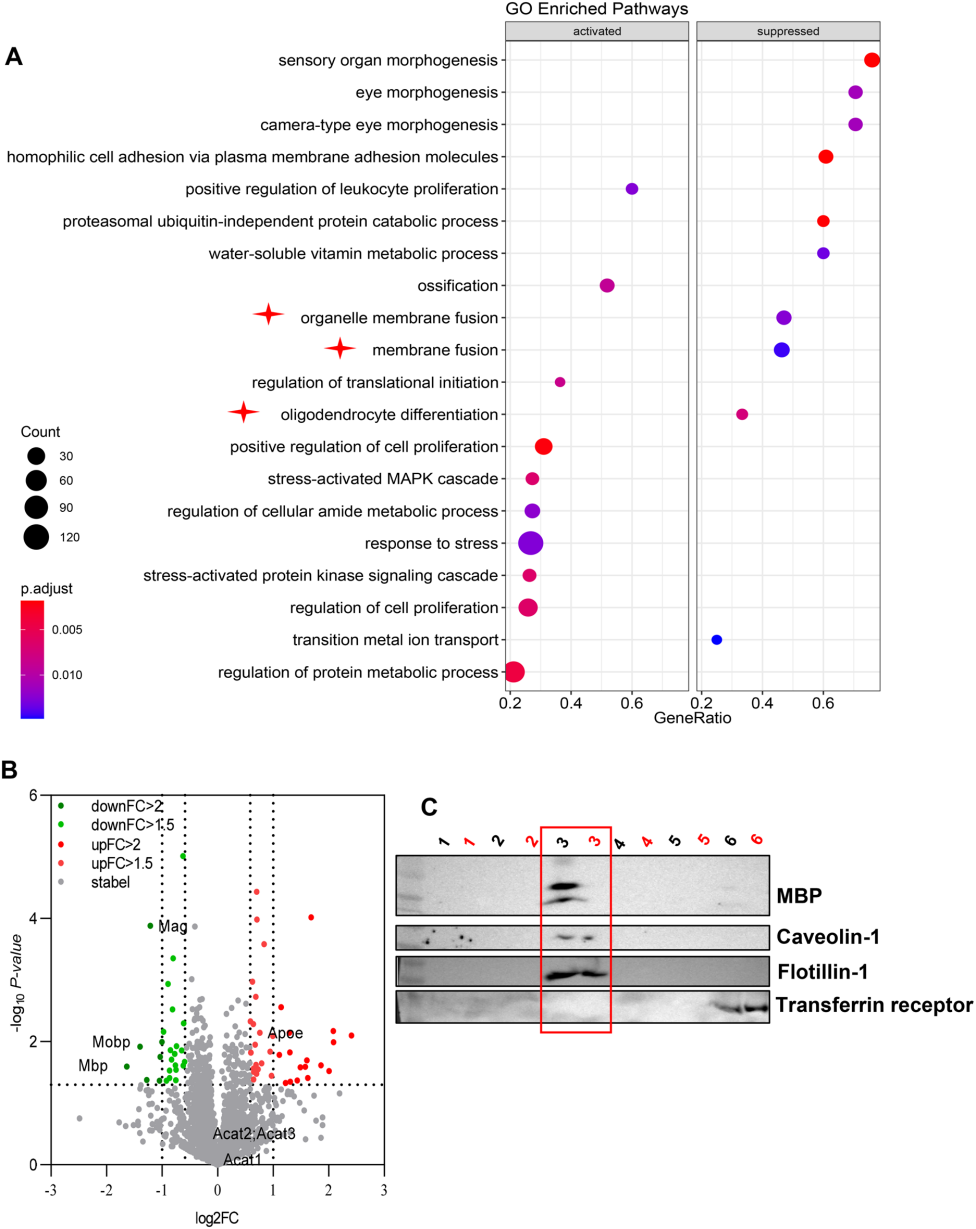
Microdomain alterations in cKO mice. (**A**) GO enrichment analysis of brain tissue proteomics of 3-month-old male cKO-nestin mice revealed that oligodendrocyte development pathway, membrane fusion pathway and organelle membrane fusion pathway were inhibited (red stars), *n* = 4 for Ctrl and cKO-nestin mice. The size and color of the dots denote the count and magnitude of significance. (**B**) Volcano plot representation of proteins with significant Fold change. Red denotes a significant 1.5-Fold or 2-Fold increase in cKO-nestin, green denotes a significant 1.5-Fold or 2-Fold decrease in cKO-nestin. (**C**) Western blot results of each layer of membrane microdomains. Numbers above each lane depict the number of layers after density gradient centrifugation. Black numbers represent Ctrl mice, and red numbers represent cKO-nestin mice. GO, gene ontology; FC, Fold-change; MBP, myelin basic protein; cKO-nestin, nervous system-specific *CerS2* knockout

To elucidate the mechanism connecting *CerS2* deficiency to myelination abnormalities, we conducted proteomics on the brains of 3-month-old male cKO-nestin mice and Ctrl mice. Gene Ontology (GO) enrichment revealed downregulation of pathways related to membrane and organelle membrane fusion (Fig. 5A and Supplementary Table 3) – which are central molecular events underlying the construction of CNS myelin sheath – in cKO-nestin mice [40]. Given the known role of sphingolipid-enriched membrane microdomains in facilitating membrane fusion in general [41], we hypothesized that deficiency in CerS2-driven production of VLC sphingolipids compromises membrane microdomain integrity, which jeopardizes membrane fusion events that underpin normal myelination in the brain. Indeed, we observed a diminished band corresponding to lipid raft fraction on density gradient centrifugation of brain homogenates from cKO-nestin mice relative to controls (Supplementary Fig. 5A). Lipidomics analyses of the purified raft fraction revealed significant depletion of VLC ceramides and their downstream sphingolipid products in cKO-nestin mice (Supplementary Fig. 5B-E and Supplementary Table 4). Given the established role of membrane microdomains as localized platform for protein-lipid and protein-protein interactions, we next examined the proteome of isolated raft fractions, revealing notable reduction in myelin basic protein (MBP) – the structural protein associated with myelin sheaths – in cKO-nestin mice (Fig. 5B). Extending this analysis to other critical myelin proteins, we observed a significant co-reduction in proteolipid protein (PLP1, the most abundant CNS myelin protein) and myelin-associated glycoprotein (MAG), whereas the level of myelin oligodendrocyte glycoprotein (MOG) was unaltered. This pattern of changes, with multiple key structural components (MBP, PLP1, MAG) being diminished, is consistent with the observed hypomyelination phenotype. Diminished raft-localization of MBP was further confirmed using immunoblot analyses of gradient fractions, showing markedly reduced MBP levels (black: Ctrl; red: cKO-nestin) under Fraction 3 corresponding to the lipid raft fraction (Fig. 5C). Our findings indicate that *CerS2* deficiency depletes VLC sphingolipids from membrane microdomains, which in turn displaces MBP – a key myelin structural protein – and leads to myelination abnormalities.

### Pharmacological disruption of membrane microdomains recapitulates myelination defects in vitro

**Fig. 6:**
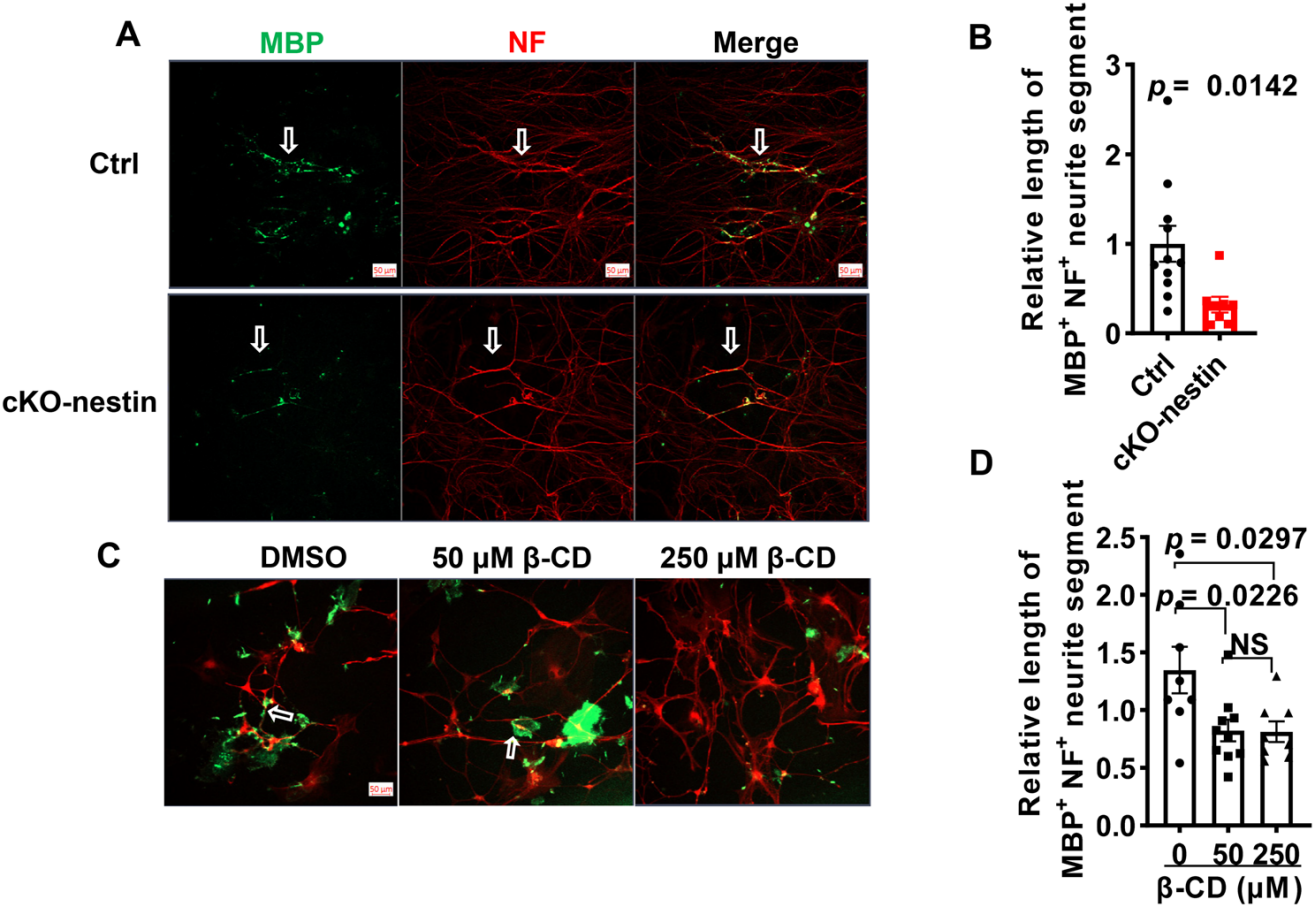
β-CD mediated effect on OL-DRG co-culture. (**A**) Immunofluorescence (IF) staining results for OL from cKO-nestin mice or Ctrl co-cultured with wild type DRG neurons, scale bar, 50 μm. (**B**) Length of myelinated axons, *n* = 11 for Ctrl, *n* = 8 for cKO-nestin mice. (**C**) IF staining results for OL-DRG after adding β-CD. DMSO (left, *n* = 8), 50 μM β-CD (middle, *n* = 10), and 250 μM β-CD (right, *n* = 8) were added to the control group, scale bar, 50 μm. (**D**) Length of myelinated axons by addition of β-CD,. The myelinated axons are indicated with white open arrows. Significance was calculated using two-tailed *t* test. OL, oligodendrocyte; DRG, dorsal root ganglion; MBP, myelin basic protein; NF, neurofilament; β-CD, β-cyclodextrin; DMSO, dimethyl sulfoxide; cKO-nestin, nervous system-specific *CerS2* knockout

We established in vitro model of myelination based on dorsal root ganglion (DRG) neuron-OPC co-culture. We first confirmed that OPCs from cKO-nestin mice produced significantly shorter myelinated segments compared to equal numbers of OPCs from Ctrl mice when co-cultured with DRG neurons (Fig. 6A–B). To directly verify the role of membrane microdomain integrity in myelination, we added β-cyclodextrin (β-CD), which disrupts membrane microdomains by depleting cholesterol, to the co-culture. Addition of 50 μM β-CD to wild-type co-cultures severely impaired myelination (Fig. 6C), and a higher dose at 250 μM showed no additive effect (Fig. 6D). These results showed that membrane microdomain disruption (from cholesterol depletion) is sufficient to impede myelin sheath formation, mirroring the myelin defects observed in CerS2-deficient OPCs.

### VLC sphingolipids determine oligodendrocyte differentiation prerequisite for effective myelination

**Fig. 7:**
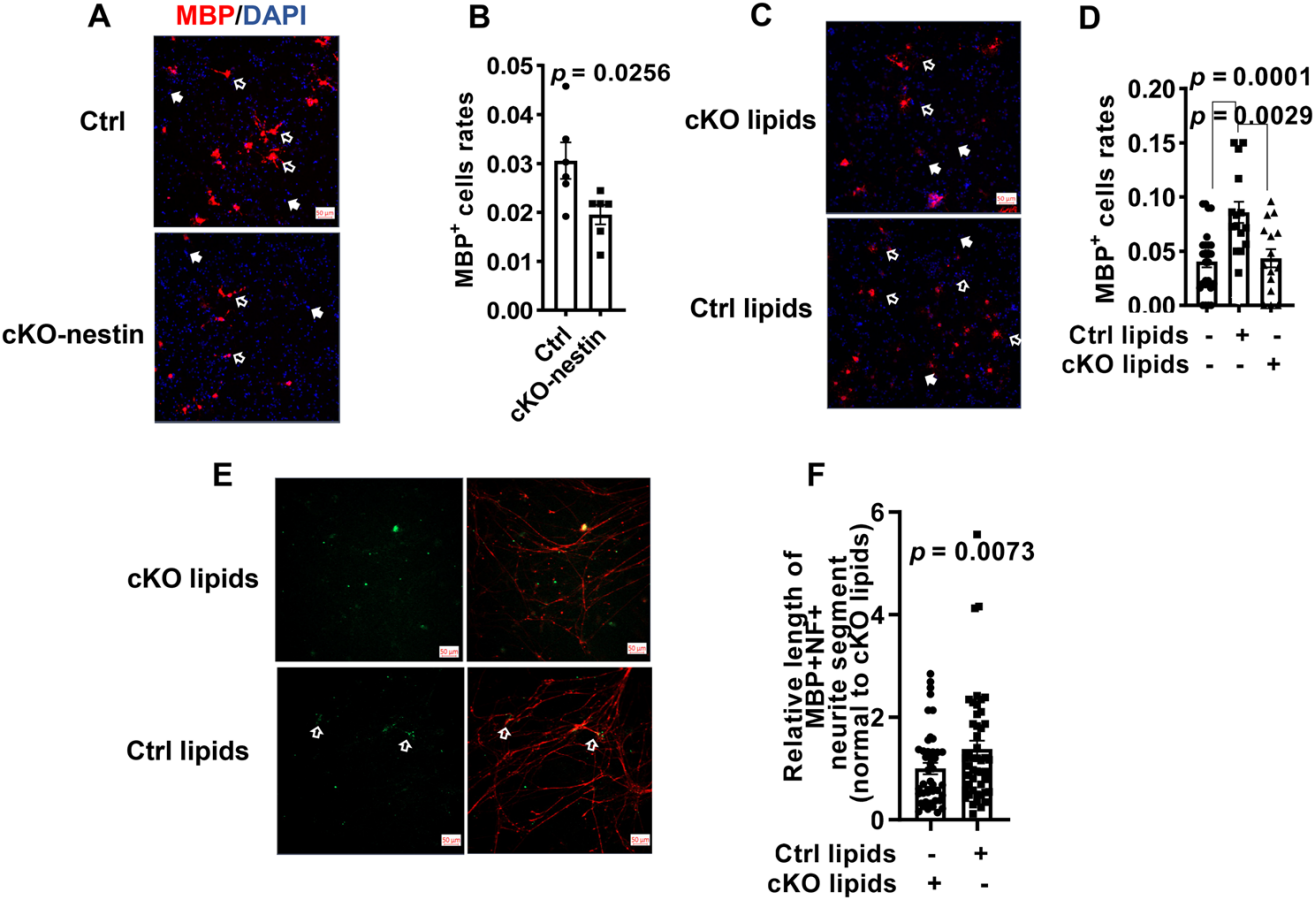
Effect of VLC sphingolipids on the OL differentiation and myelination. (**A**) IF staining of oligodendrocytes differentiation in vitro from cKO-nestin mice or control. The OL are indicated with white open arrows scale bar, and white closed arrows indicate other cells, 50 μm. (**B**) Differentiation rates of oligodendrocytes from cKO-nestin mice and Ctrl in vitro, *n* = 5. (**C**) IF results of differentiated OL from cKO-nestin mice supplemented with 10 μM lipids obtained from the brain of cKO-nestin mice or control. The OL are indicated with white open arrows scale bar, and white closed arrows indicate other cells, scale bar, 50 μm, and (**D**) Differentiation rates of OL in the presence (*n* = 29) or absence of WT lipids and cKO lipids, *n* = 15. (**E**), IF staining of cKO OL-DRG co-culture results by supplemented with 10 μM lipids obtained from the brain of cKO-nestin mice or Ctrl in vitro. The myelinated axons are indicated with white open arrows, scale bar, 50 μm, and (**F**) Length of myelination axons, *n* = 45 for cKO-nestin lipids, *n* = 47 for Ctrl lipids. IF, immunofluorescence; MBP, myelin basic protein; OL, oligodendrocyte; cKO-nestin, nervous system-specific *CerS2* knockout

Brain proteomics indicated downregulated oligodendrocyte differentiation in cKO-nestin mice (Fig. 5A). To functionally validate this, we performed an in vitro differentiation assay, which revealed that the proportion of mature oligodendrocytes (measured by MBP+ cells) from cKO-derived neural progenitor cells was significantly lower than that from Ctrl (Fig. 7A–B). This confirmed that *CerS2* deletion intrinsically impairs oligodendrocyte generation. Since lipidomics indicated massive reductions in VLC sphingiolipids as the primary biochemical defects of cKO-nestin brain, we sought to determine if these lipids were functionally responsible for the developmental deficit in oligodendrocytes. We supplemented the culture medium of cKO-derived neural progenitor cells with lipid extracts from the brain tissues of Ctrl and cKO-nestin mice, respectively, and co-culture the developing oligodendrocytes with DRG neurons in vitro. Strikingly, supplementation of Ctrl-derived lipids significantly increased the proportion of mature oligodendrocytes (Fig. 7C–D) and enhanced myelination in the DRG co-culture system (Fig. 7E–F). In contrast, lipid extracts from cKO-nestin brains, deficient in VLC sphingolipids, failed to produce these effects.

Among the altered lipids, we also noted significantly reduced levels of S1P in the brain tissue of cKO-nestin mice (Fig. 3E and Supplementary Fig. 3A). Direct addition of S1P to cKO-derived neural progenitor cells, however, did not rescue impaired oligodendrocyte differentiation (Supplementary Fig. 6A-B). These results rule out S1P deficiency as primarily responsible for the observed phenotypes, and further underscore the specific and essential role of VLC sphingolipids in oligodendrocyte maturation that underlies normal myelination. The failure of cKO-derived lipid extracts (which lack VLC sphingolipids) to replicate the rescue effect provides further evidence that the active components within the wild-type extract are indeed CerS2-dependent VLC sphingolipids. We acknowledge that a definitive causal link requires supplementation with purified individual VLC species, but this remains technically challenging due to difficulties in isolating specific VLC sphingolipids (e.g., distinct ceramides). As an initial step, we supplemented cultures with C24:0 sphingomyelin (SM24), a representative VLC sphingolipid, and observed a partial positive effect on oligodendrocyte differentiation (Supplementary Fig. 6C-D), consistent with a role for VLC sphingolipids in this process.

## Discussion

The biological significance of lipid metabolites, particularly in the lipid-rich milieu of the brain, has been increasingly recognized [42–45]. Disruption in lipid homeostasis is implicated in the pathogenesis of a myriad of neurological diseases [46–48], and coordinated lipid metabolism is crucial for normal brain function [32, 36, 49, 50]. In this study, we systematically demonstrate that deficiency in brain VLC sphingolipids resulting from *CerS2* deletion plays a primary role in severe myelin defects. We first established that CNS-specific deletion of *CerS2* (cKO-nestin) recapitulates multiple aspects of neurological diseases, including weight loss, convulsions, increased mortality, and myelin abnormalities. Given the high expression of CerS2 in oligodendrocytes [51] – the primary myelin-producing cells of the CNS – we further generated oligodendrocyte-specific *CerS2* knockout mice (*CerS2*(OL)-cKO). These mice faithfully recapitulated the core phenotypes of cKO-nestin mice, confirming that defects in myelination are direct, cell-autonomous consequence of *CerS2* deficiency in oligodendrocytes.

CerS2 specifically generates VLC ceramides (C22–C24), which serve as the precursors for a spectrum of other VLC sphingolipids. As expected, CerS2 ablation led to pronounced reductions in VLC ceramides and their downstream sphingolipids, including SL, GluCer, GalCer and SM, with compensatory increases in shorter-chain sphingolipids (C16–C18) (Figs. 1A and 3A-E). The precise fatty acyl chain lengths of ceramides and downstream complex sphingolipids critically determine their biological function [13], and are particularly important for sustaining normal neural function [52–54]. Our data substantiate that VLC sphingolipids are indispensable for normal brain development, and that compensatory increases in shorter chain sphingolipids in the context of *CerS2* deficiency cannot rescue developmental defects, particularly pertaining to myelination. MSI also supported the functional non-redundancy between VLC sphingolipids and their shorter-chain counterparts, which displayed distinct spatial enrichment in brain cross-sections. In particular, we observed strong colocalization between regions of deficient CerS2-derived VLC sphingolipids and the sites of myelin disruption, highlighting the functional primacy of CerS2-dependent lipid metabolism in maintaining overall myelination (Fig. 4).

The critical involvement of VLC sphingolipids in myelination was further demonstrated in rescue experiments leveraging an in vitro model of myelination, wherein lipid extracts from the brains of wild-type mice, but not cKO-nestin mice, restored oligodendrocyte differentiation and myelination in co-cultures of DRG neurons with OPCs from cKO-nestin mice (Fig. 7C–F). While S1Ps were also present in wild type brain lipid extracts, their supplementation alone failed to rescue the myelin-related defects, highlighting the critical and specific requirement for VLC sphingolipids in oligodendrocyte differentiation and myelination. Our lipidomics analyses of cKO-nestin brains revealed compensatory increases in C16 ceramides that are clinically relevant. Specifically, elevated C16 ceramides had been documented in the brain and plasma of patients suffering from MS, and neuron-specific deletion of both *CerS5* and *CerS6*, which mediate C16–C18 ceramide biosynthesis, was shown to be neuroprotective in an experimental model of MS [55]. Based on these evidence, we surmise that *CerS2* knockout creates a double-hit scenario – loss of protective VLC sphingolipids and accretion of potentially detrimental shorter-chain ceramides – that critically contributes to severe myelination defects.

Despite the global lipid remodeling observed upon *CerS2* knockout, the mRNA levels of related lipid biosynthetic enzymes, such as UGCG, SPHK1 and DGKA, were not altered in the brain of cKO-nestin mice. Proteomics analyses of the brain similarly did not uncover perturbed regulation of lipid metabolic pathways (Fig. 5A). These findings suggest that pathological lipid remodeling in the context of *CerS2* deficiency was not driven by transcriptional reprogramming, but rather, by a fundamental shift in substrate availability. Ablation of CerS2, the critical gatekeeper enzyme modulating precursor substrate bioavailability for downstream sphingolipid production, skews the ceramide pool away from VLC species toward shorter-chain (C16–C18) species. Thus, the global lipid remodeling in cKO-nestin mice primarily stems from post-translational perturbation of metabolic flux arising from an unbalanced pool of ceramide precursors.

Proteomics of the brain uncovered downregulation of plasma membrane fusion and organelle membrane fusion pathways in cKO-nestin mice, which is likely associated with the loss of VLC sphingolipid-enriched microdomains [56]. Loss of VLC sphingolipids from lipid rafts, which likely serve as a platform for organizing myelin proteins such as MBP, critically compromises the process of myelination. Our finding that pharmacological disruption of membrane microdomains with β-CD potently inhibits myelination in vitro provides further support for this model. Based on these observations, we hypothesize that VLC sphingolipids contribute to the establishment of thick, ordered lipid bilayers that confer the optimal hydrophobic microenvironment for the stable integration and anchoring of MBP, since MBP contains hydrophobic domains capable of inserting into lipid bilayers [57]. The stable anchorage of MBP within VLC sphingolipid-enriched microdomains is an important prerequisite for compact myelin assembly.

Apart from being critical structural determinants of the optimal membrane microenvironment, VLC sphingolipid-enriched microdomains is also important for brain development by facilitating processes such as synaptic transmission, signal transduction, as well as membrane assembly [20, 58, 59]. This highlights the dual roles of VLC sphingolipids as both structural components and signaling lipids in ensuring normal myelination and neural integrity. While our current data highlight the collective importance of VLC sphingolipids, future studies are required to dissect the specific roles of individual sphingolipid classes (e.g. VLC-SM, VLC-GluCer, VLC-GlaCer, and VLC- SL).

The pathological cascade elucidated here, stemming from a developmental deficit in CerS2, provides critical insights into the etiology of genetically-driven, early-onset myelin disorders. It is noteworthy that an inducible, adult-stage knockout of CerS2 could potentially model different aspects of demyelination, such as the progressive nature seen in multiple sclerosis (MS). While such a model would be invaluable for deciphering the mechanisms of myelin maintenance and repair in the mature nervous system, our constitutive knockout model is uniquely positioned to dissect the consequences of a developmental genetic lesion. The severe myelin defects observed as early as postnatal day 20 in our model underscore that the loss of VLC sphingolipids during critical developmental windows is sufficient to disrupt myelination fundamentally. This aligns with human pathologies where CerS2 mutations cause progressive myoclonic epilepsy, emphasizing the indispensable role of CerS2-dependent lipid metabolism in myelin development.

Furthermore, while our behavioral tests correlate with the structural myelin defects, future studies employing in vivo or slice electrophysiology will be essential to definitively establish the causal conduction deficits resulting from hypomyelination, thereby linking structural defects to functional impairment. Therefore, our findings not only establish a causative link between CerS2 deficiency and developmental myelin failure but also lay the essential groundwork for future studies investigating the potential of VLC sphingolipid supplementation as a therapeutic strategy for related neurological diseases.

In summary, we report a clear pathogenic cascade whereby CerS2 deficiency in oligodendrocytes depletes VLC sphingolipids, leading to the disruption of specialized membrane microdomains and the subsequent displacement of MBP from lipid rafts, thereby impeding proper myelination within the brain. Our work also establishes that VLC sphingolipids promote oligodendrocyte differentiation and maturation, which is essential for proper myelination to ensue. Our findings provide new insights into demyelinating pathologies, and identify the CerS2-VLC sphingolipid axis as a potential target for therapeutic intervention against neurological diseases with myelination abnormalities.

## Electronic supplementary material

Below is the link to the electronic supplementary material.

Supplementary material 1

Supplementary material 2

Supplementary Table 1

Supplementary Table 2

Supplementary Table 3

Supplementary Table 4

Supplementary video 1

## Data Availability

Source datasets underlying individual display items in the manuscript are included in supplementary tables . Other data supporting the findings of this study are available from the corresponding author on reasonable request.
