# Supplementary material for: Very long chain sphingolipids govern brain myelination by regulating oligodendrocyte differentiation and membrane microdomain integrity
Source: J Transl Med. 2026 Feb 19;24:550. doi: 10.1186/s12967-026-07881-0 (PMC13094249; doi:10.1186/s12967-026-07881-0)

Fig. 1 | Pathological changes and myelination abnormalities in cKO-nestin mice.

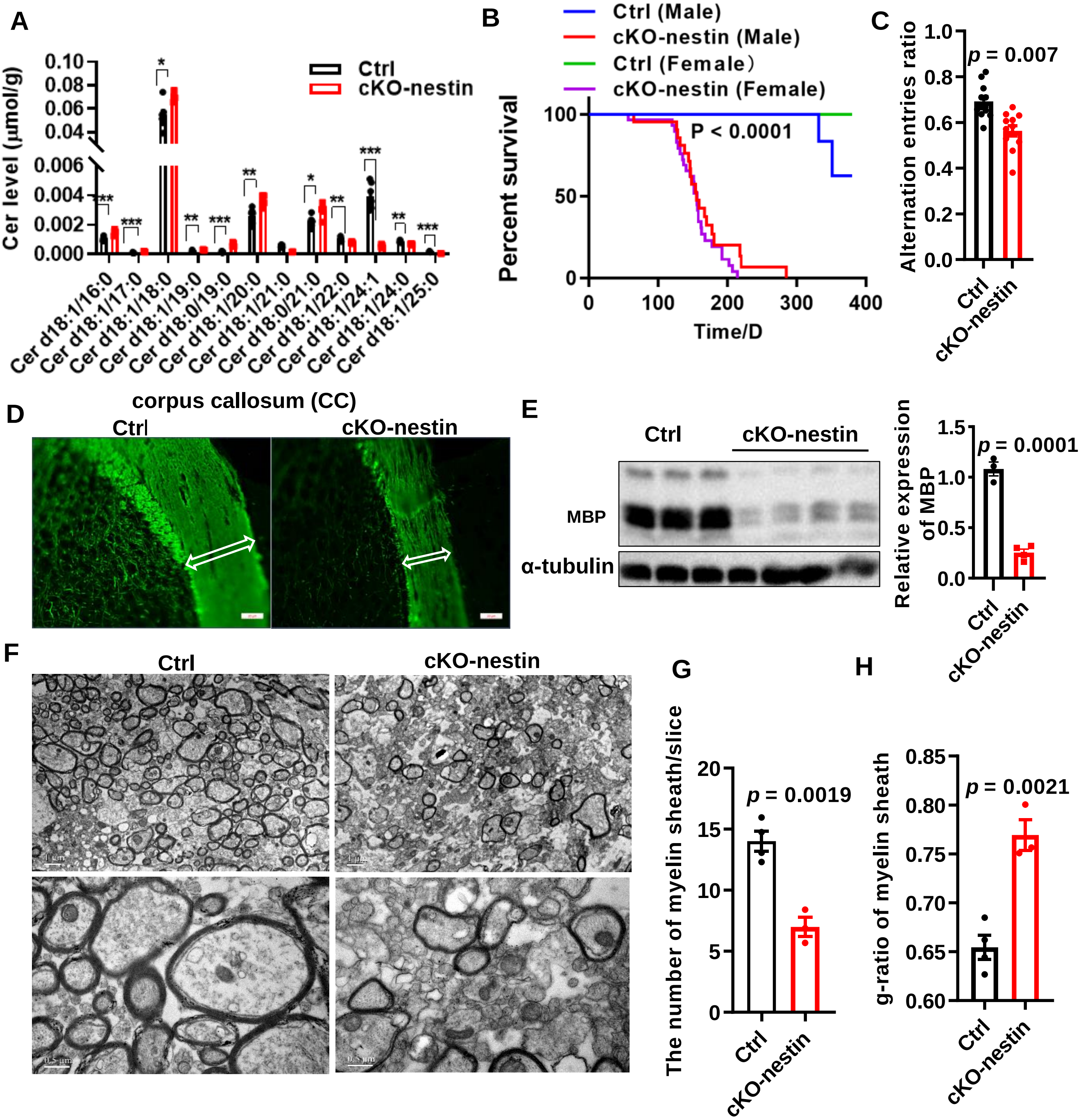

Fig. 2 | EM examination of myelination abnormalities in cKO-nestin and cKO-OL mice.

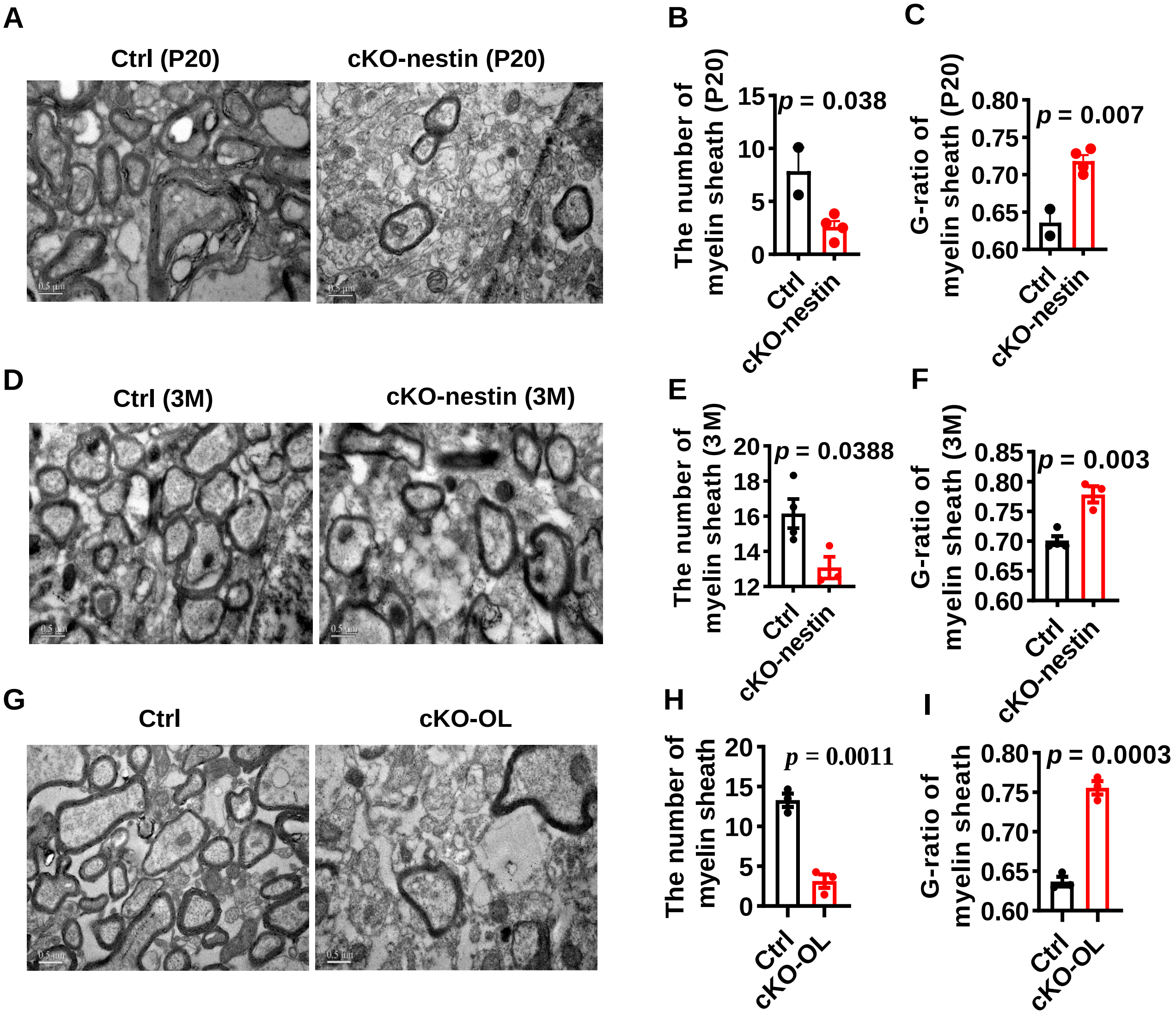

Fig. 3 | Lipid aberrations in cKO-nestin mice.

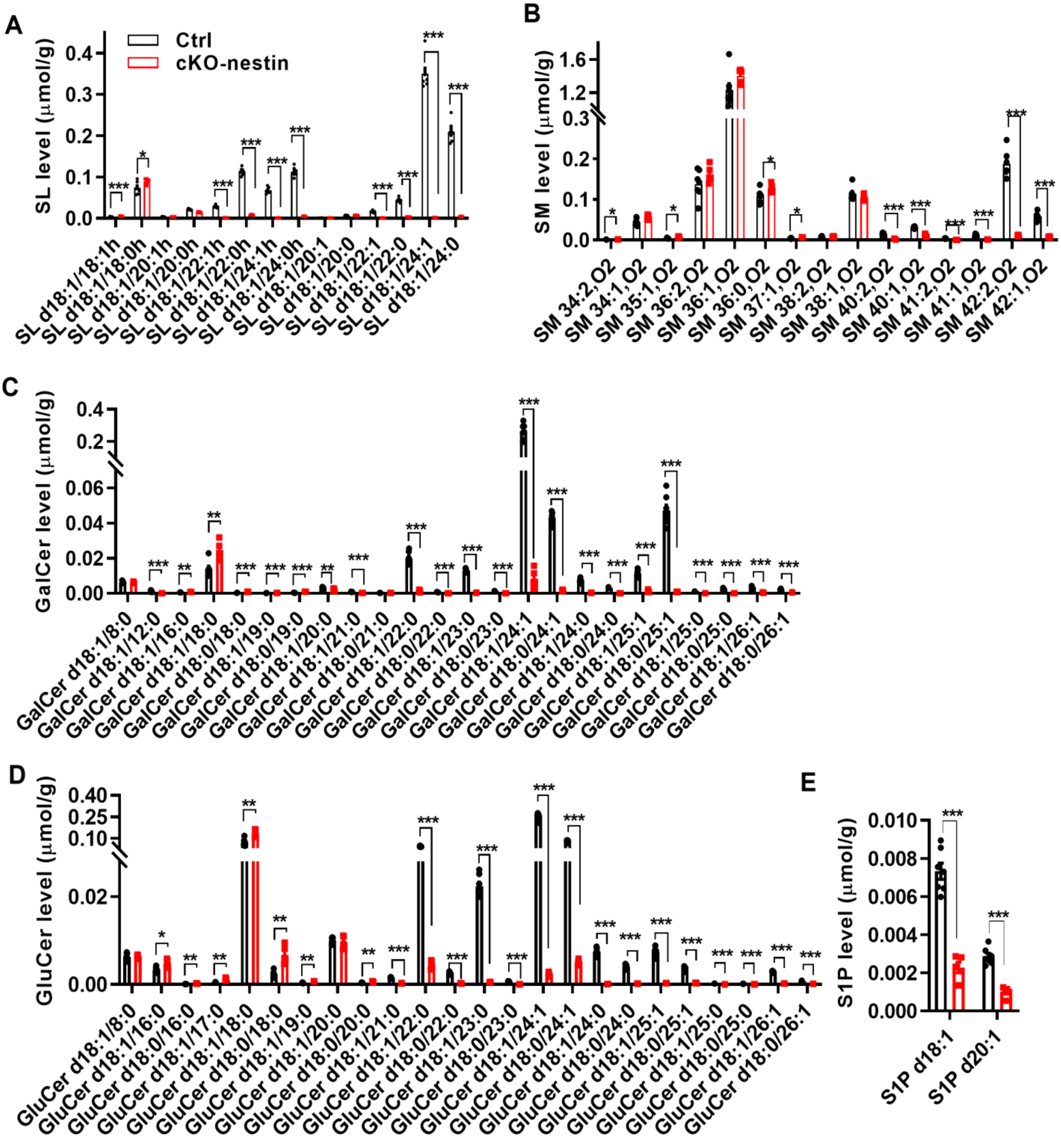

Fig. 4 | DESI-MSI of spatial lipid distribution across brain regions between Ctrl and cKO-nestin mice.

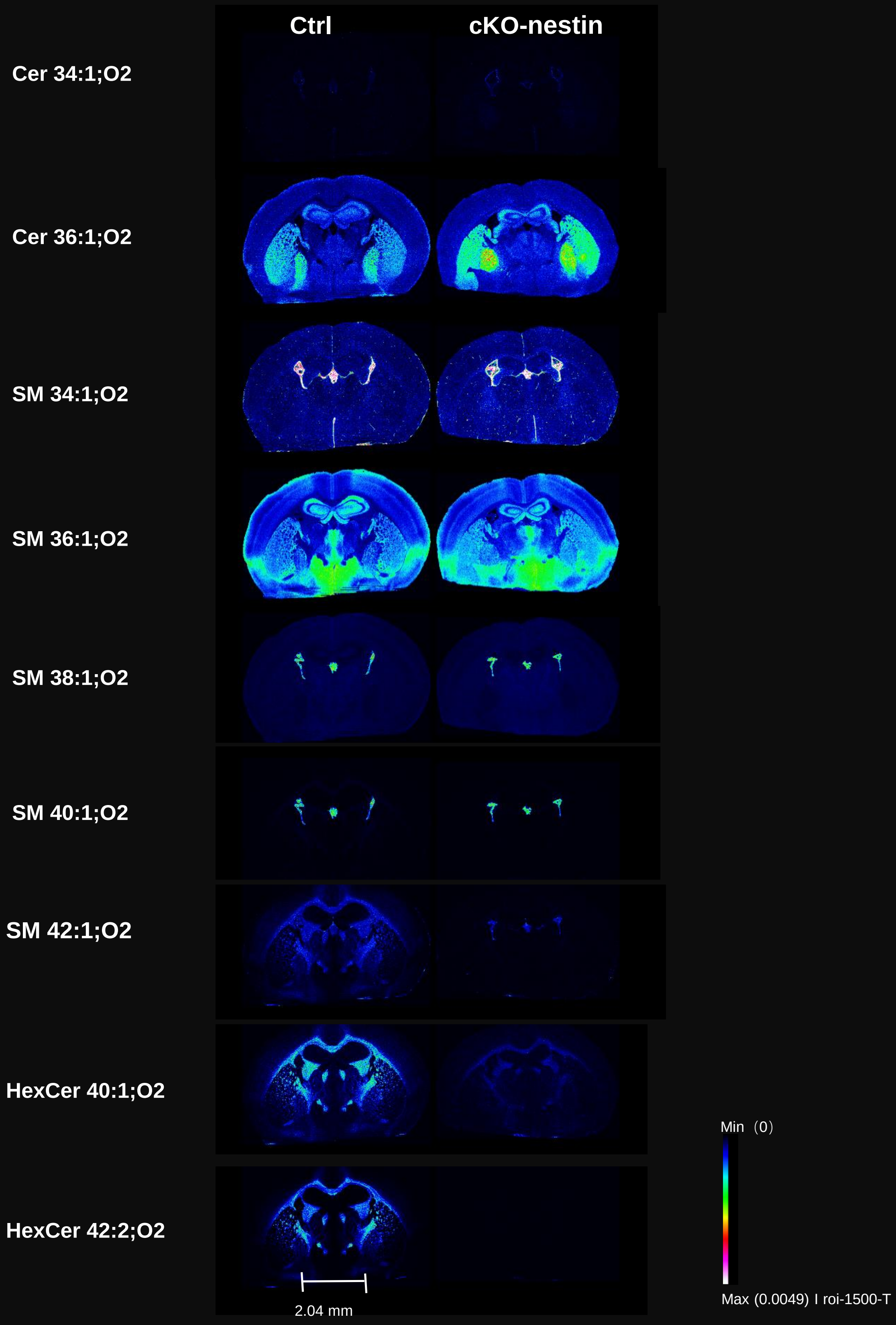

Fig. 5 | Microdomain alterations in cKO mice.

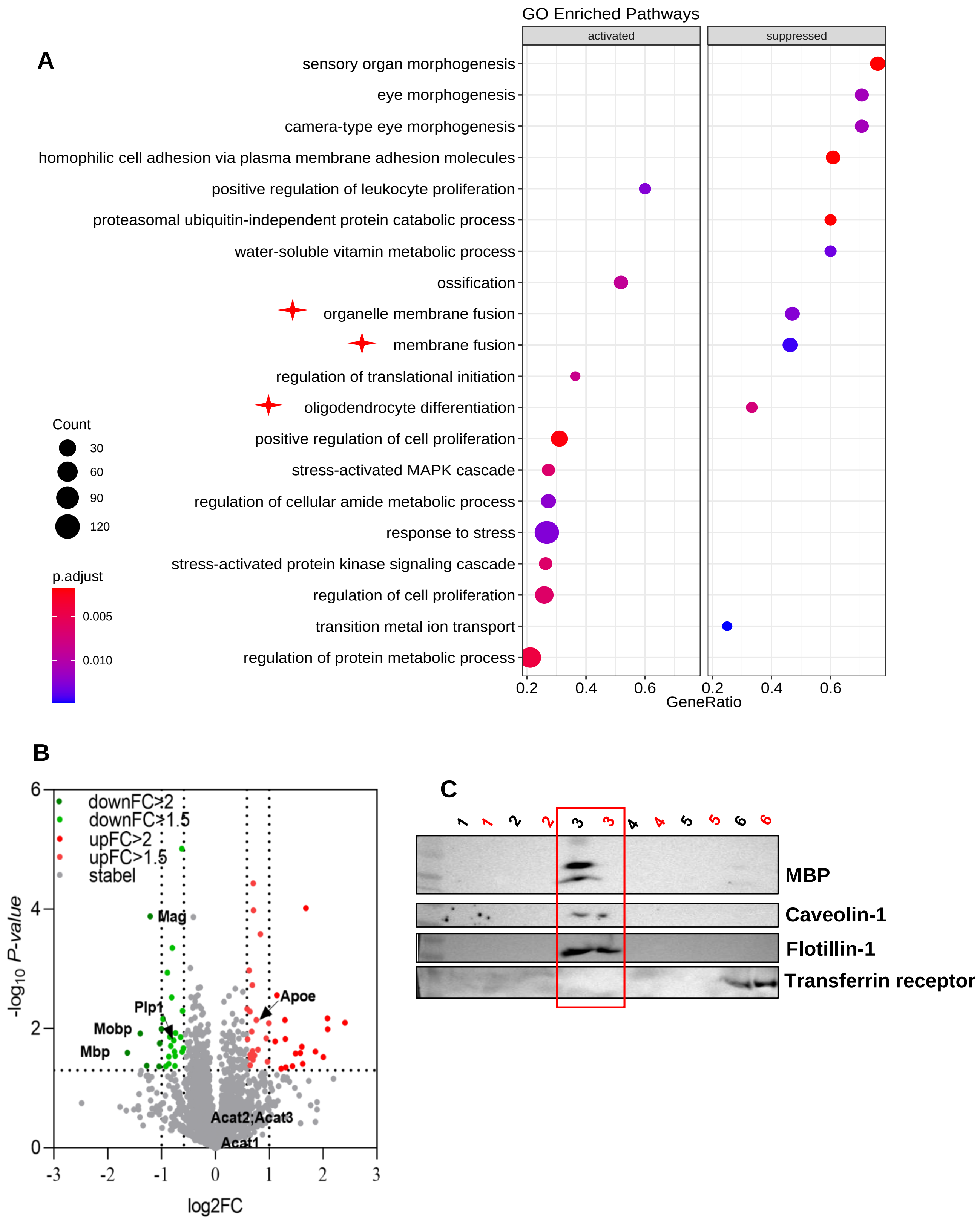

Fig. 6 | β-CD mediated effect on OL-DRG co-culture.

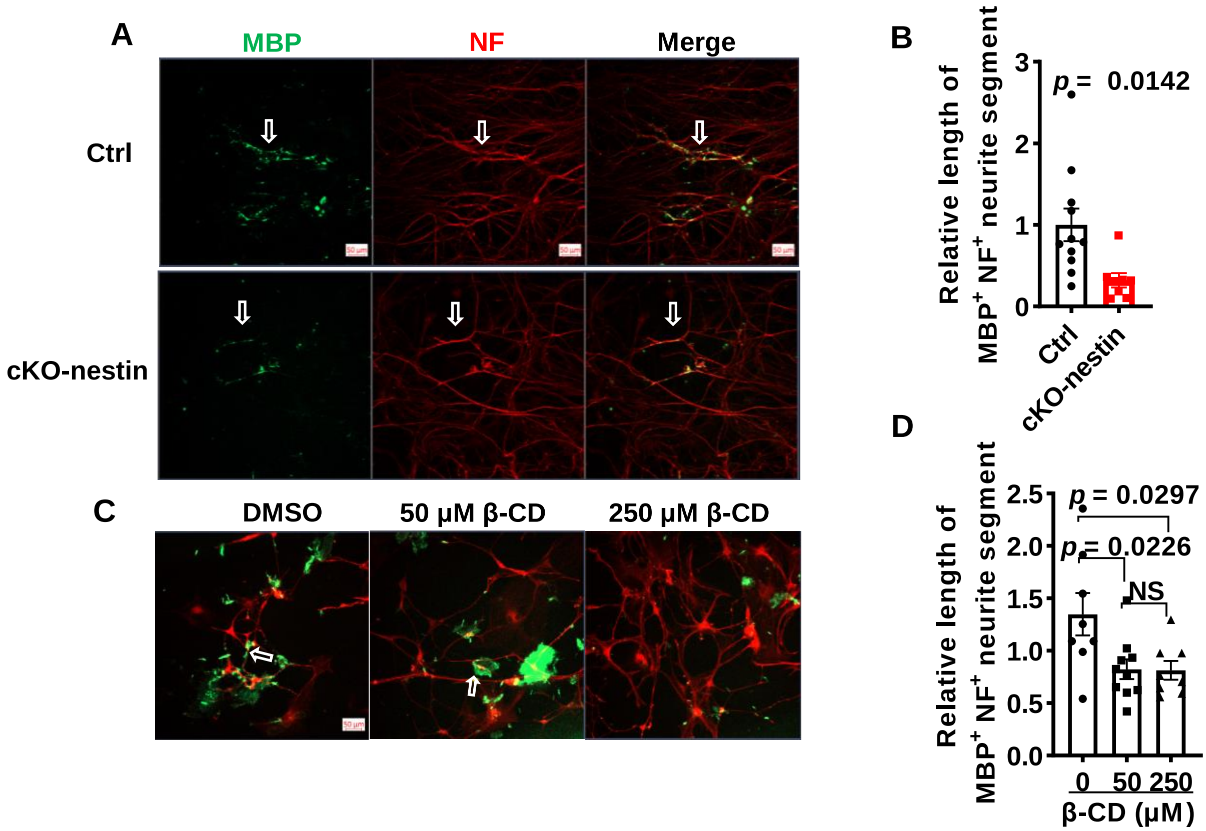

Fig.7 | Effect of VLC sphingolipids on the OL differentiation and myelination

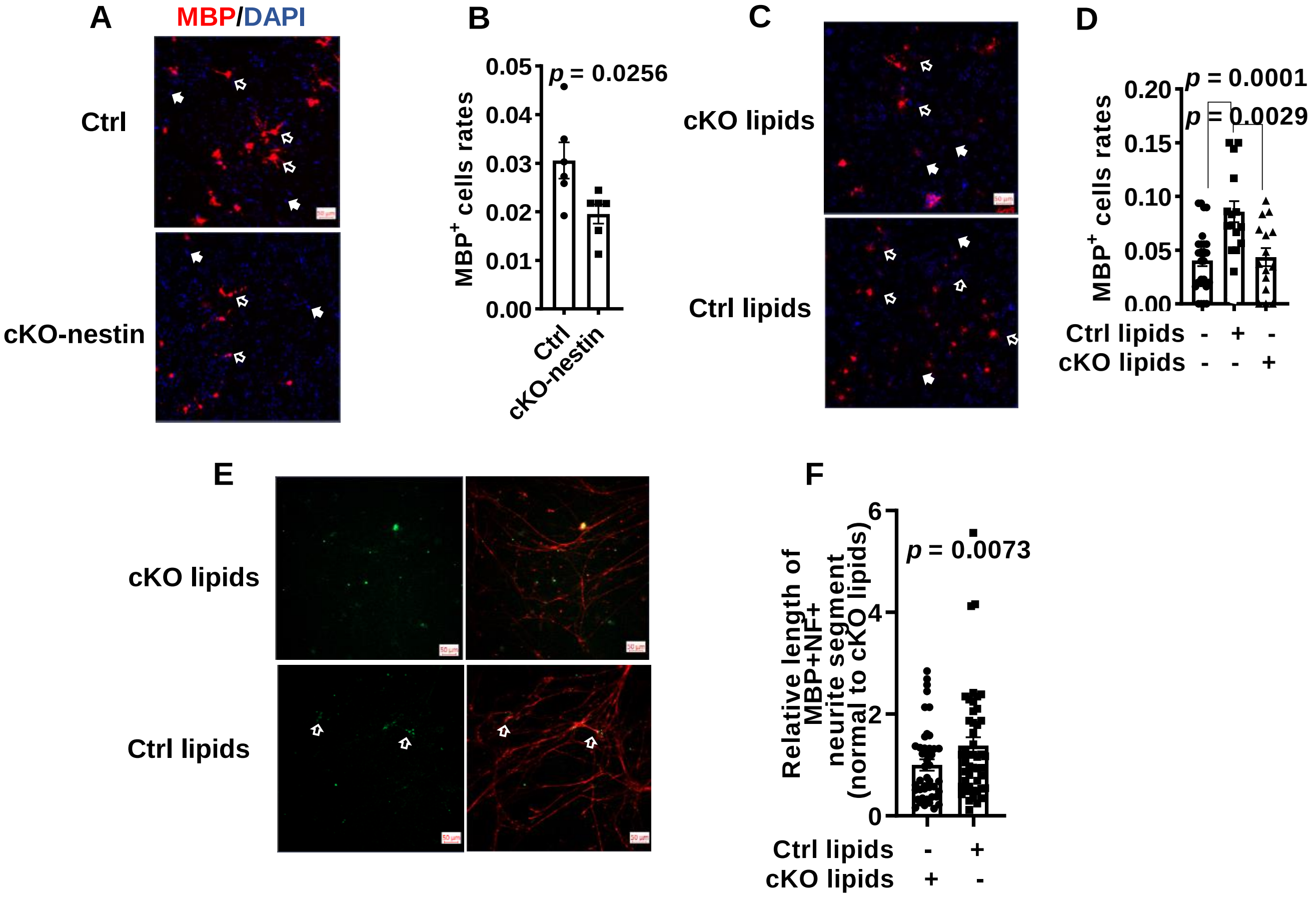

Supplementary Fig. 1 | Identification of CerS2 knock out and cKO-nestin mice abnormalities

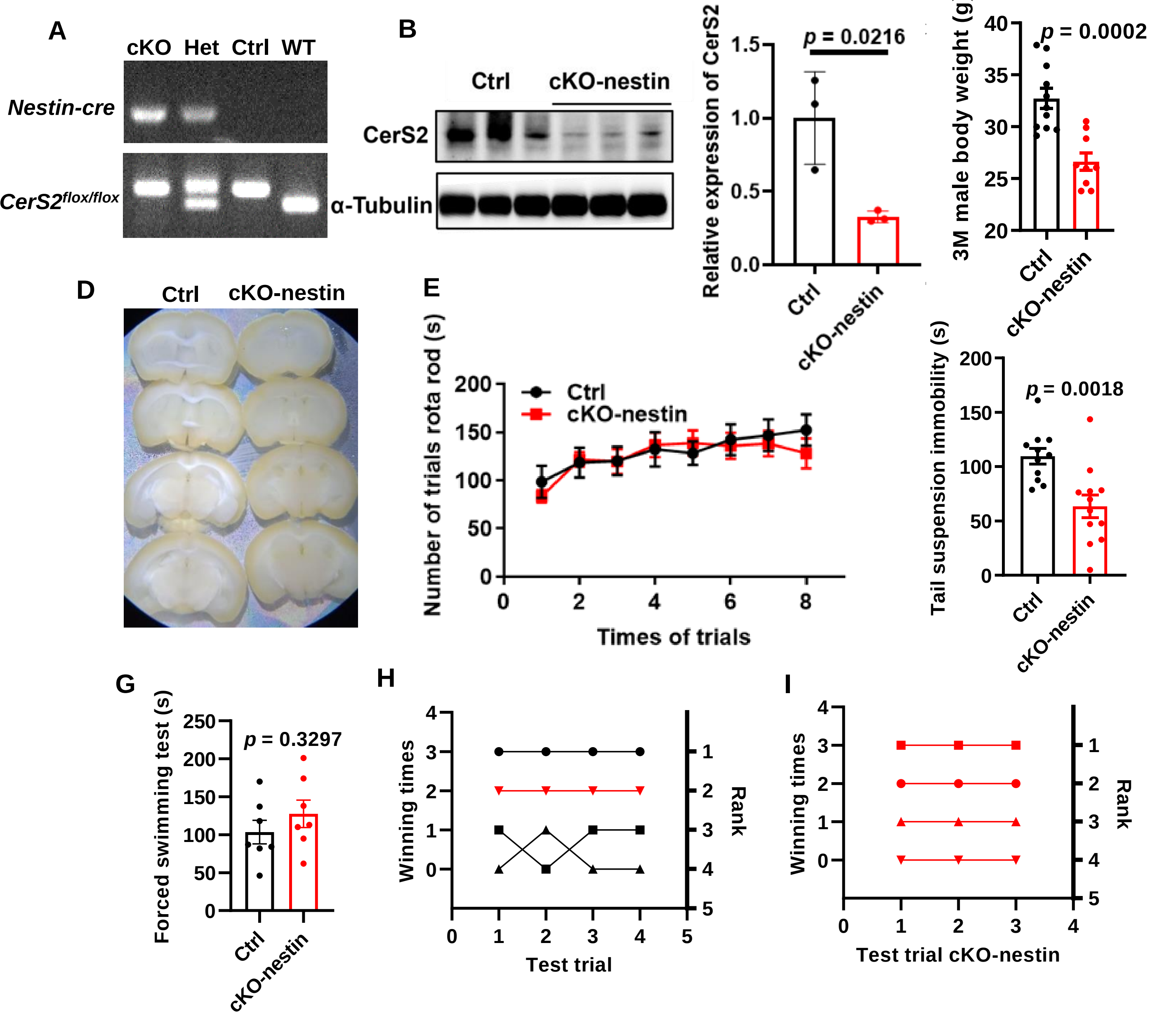

Supplementary Fig. 2 | cKO-nestin and cKO-OL mice abnormalities

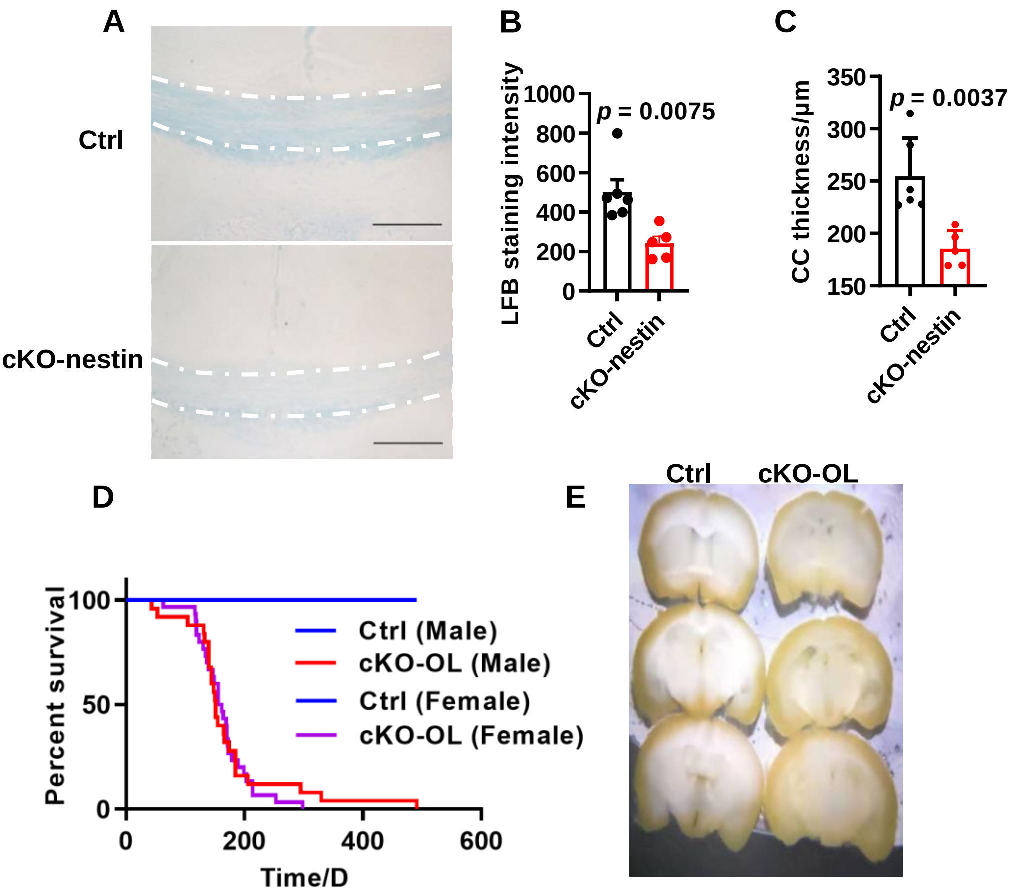

Supplementary Fig. 3 | Altered lipids and its mRNA expression levels in cKO-nestin mice.

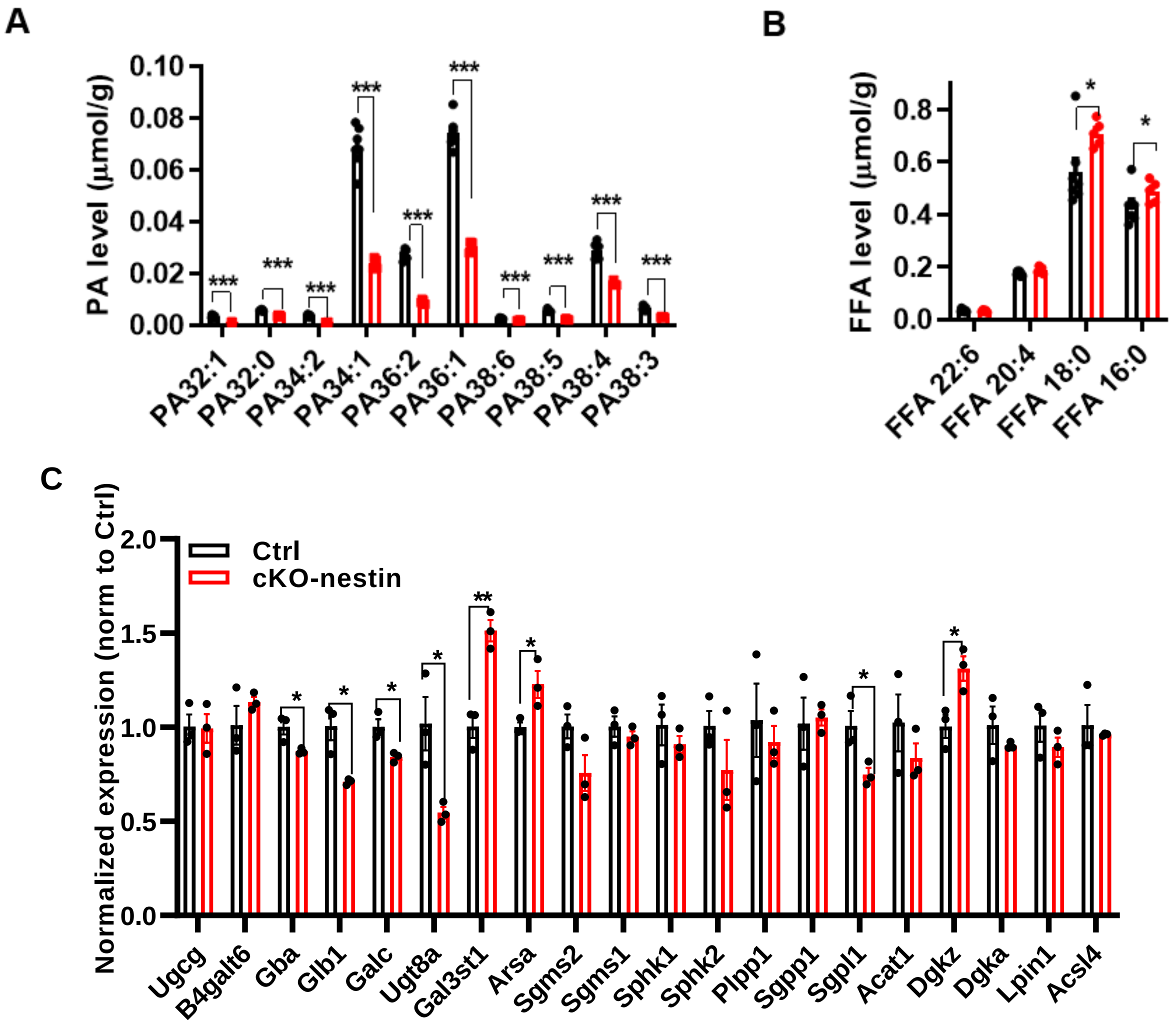

Supplementary Fig. 4 | MALDI-MSI of spatial lipid distribution across brain regions between Ctrl and cKO-nestin mice.

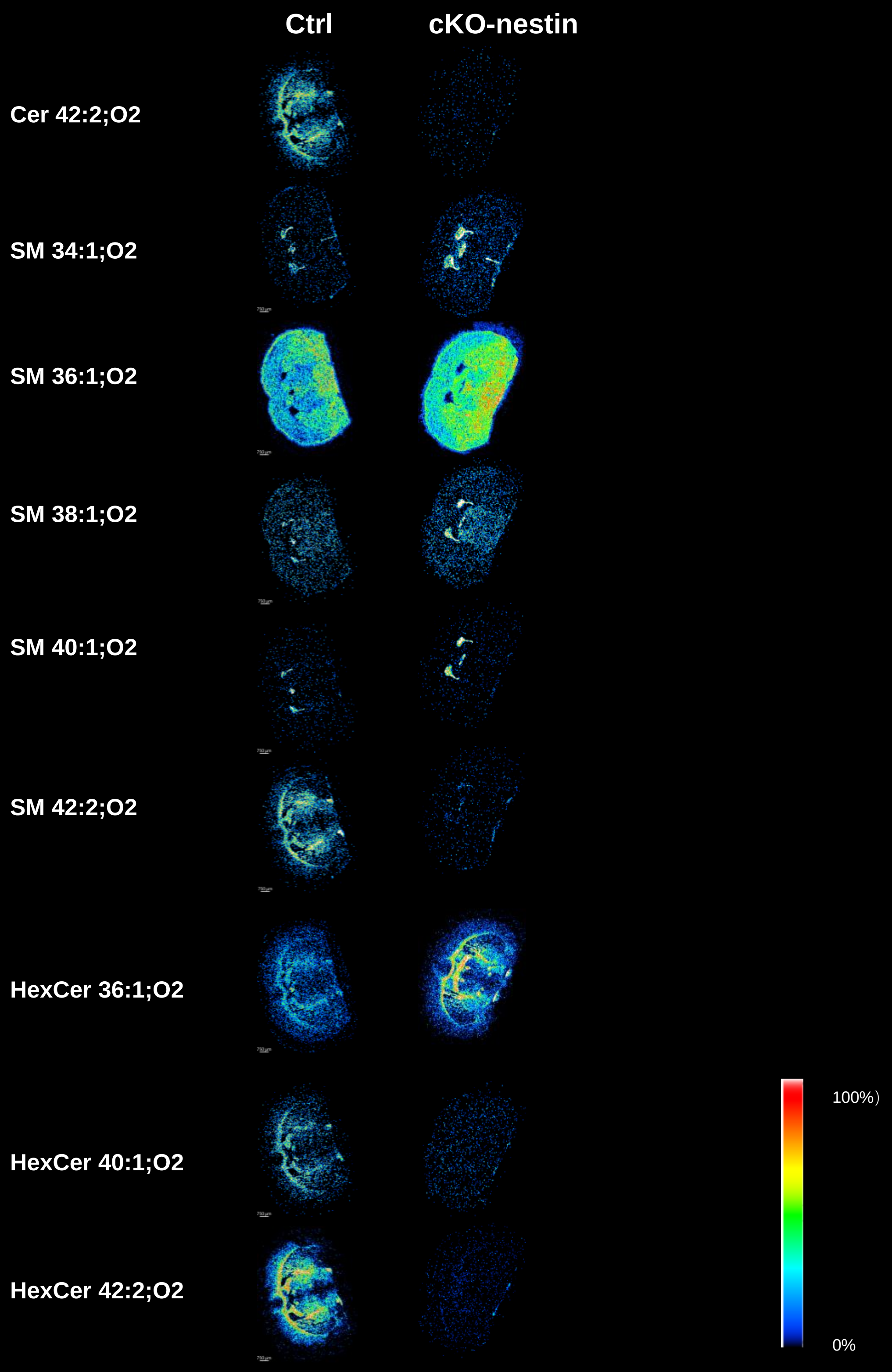

Supplementary Fig.5 | Lipid alterations in microdomains.

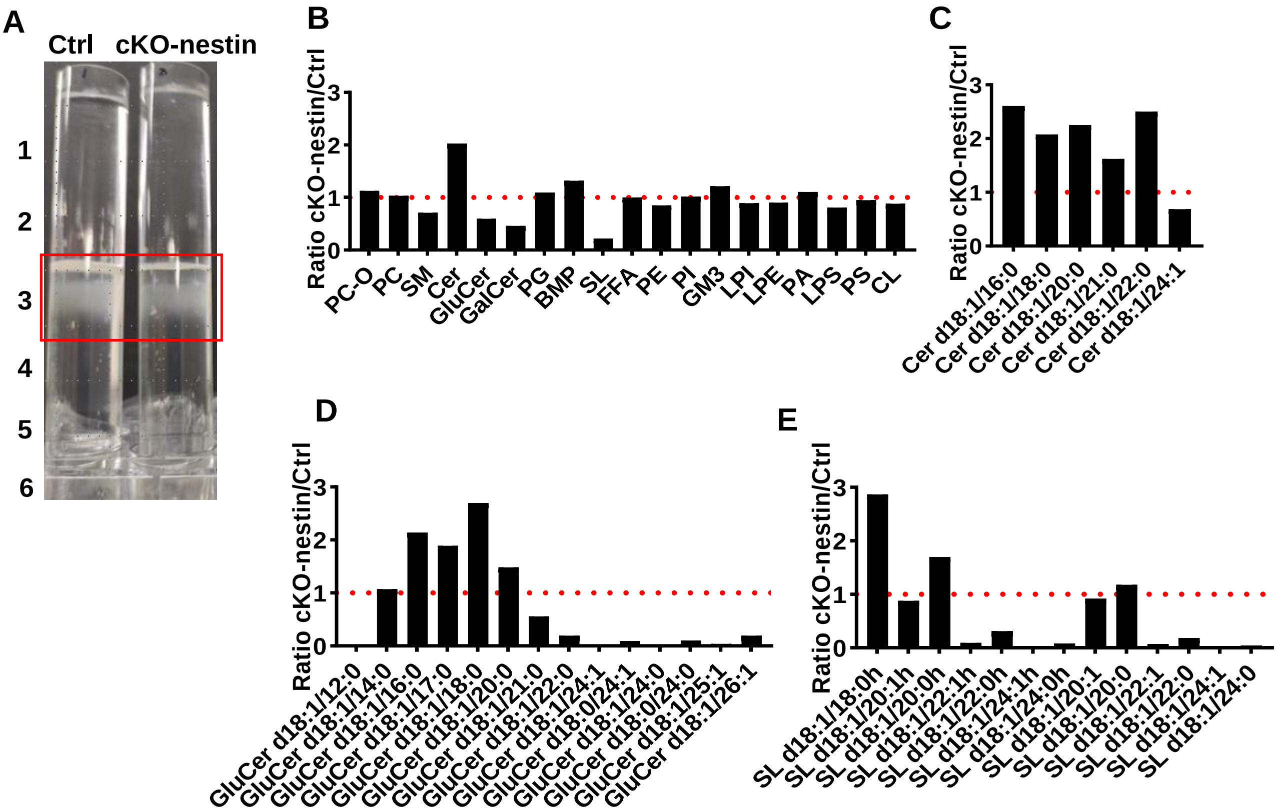

Supplementary Fig. 6 | Effect of S1P and SM 24:0:O2 on the OL differentiation.

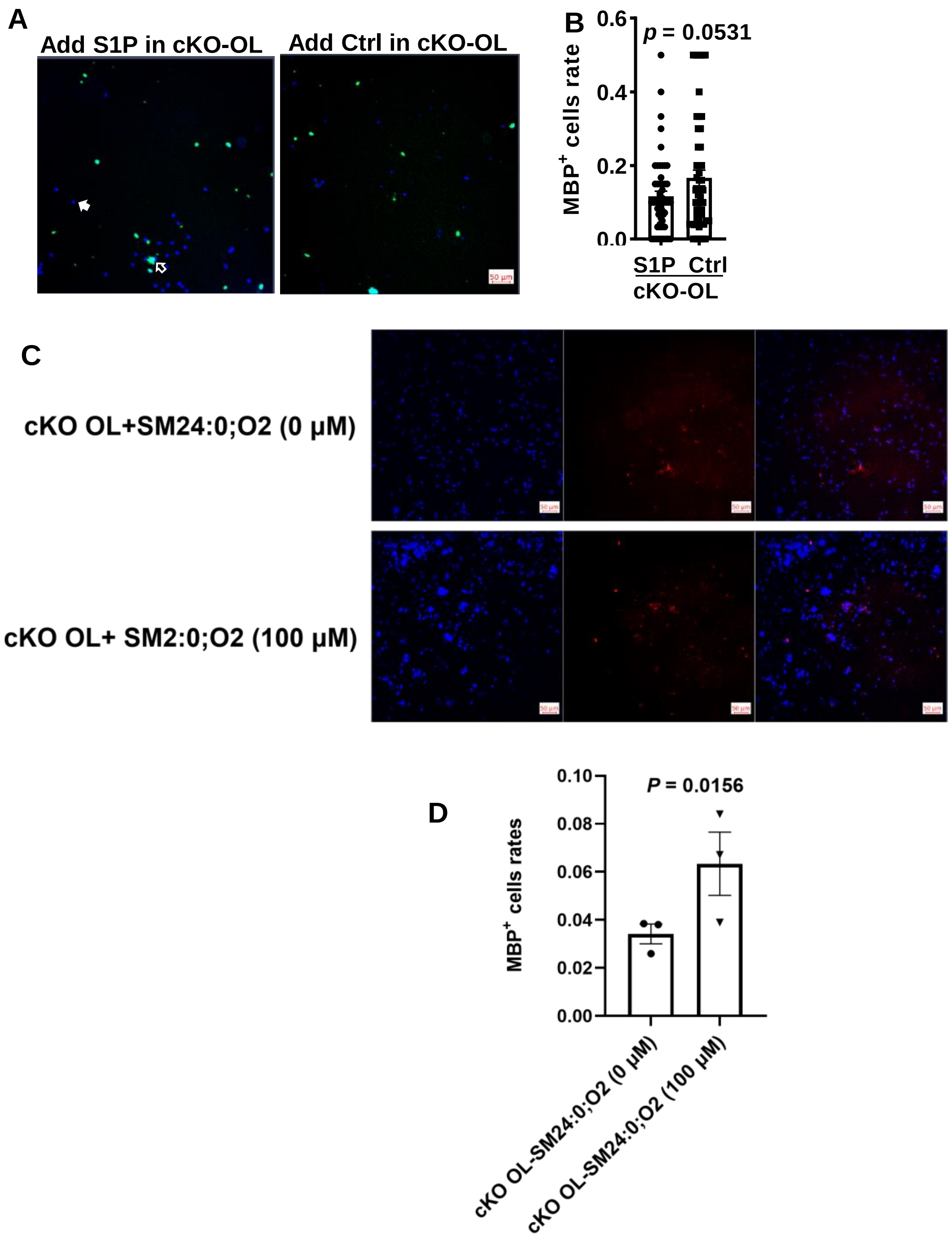

Supplement: Supplementary file 1 — Supplementary material 1 [file 12967_2026_7881_MOESM1_ESM.pdf]
