# Supplementary figures and images for: Very long chain sphingolipids govern brain myelination by regulating oligodendrocyte differentiation and membrane microdomain integrity
Source: J Transl Med. 2026 Feb 19;24:550. doi: 10.1186/s12967-026-07881-0 (PMC13094249; doi:10.1186/s12967-026-07881-0)

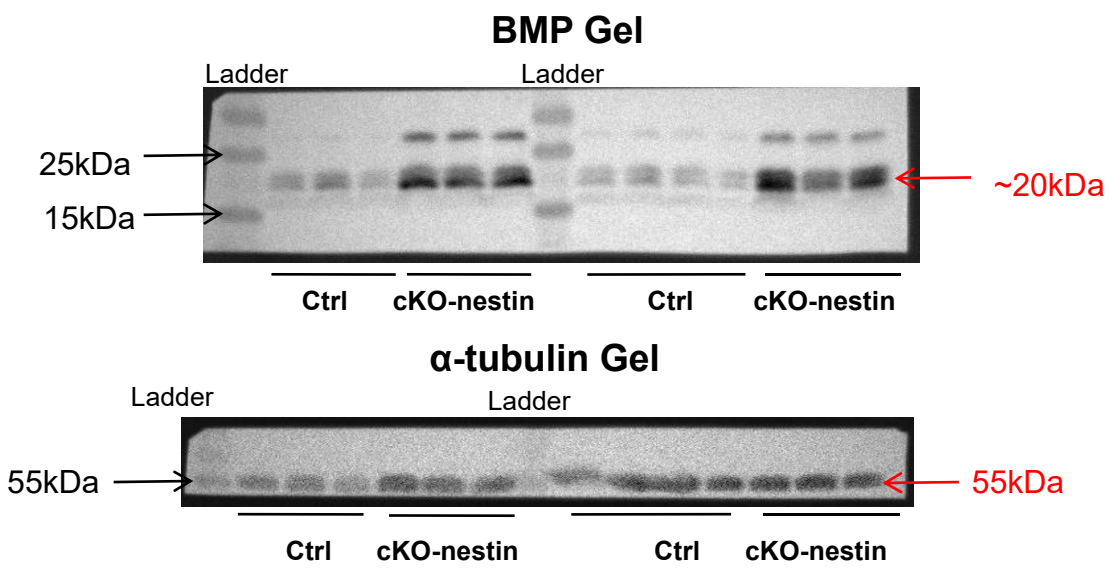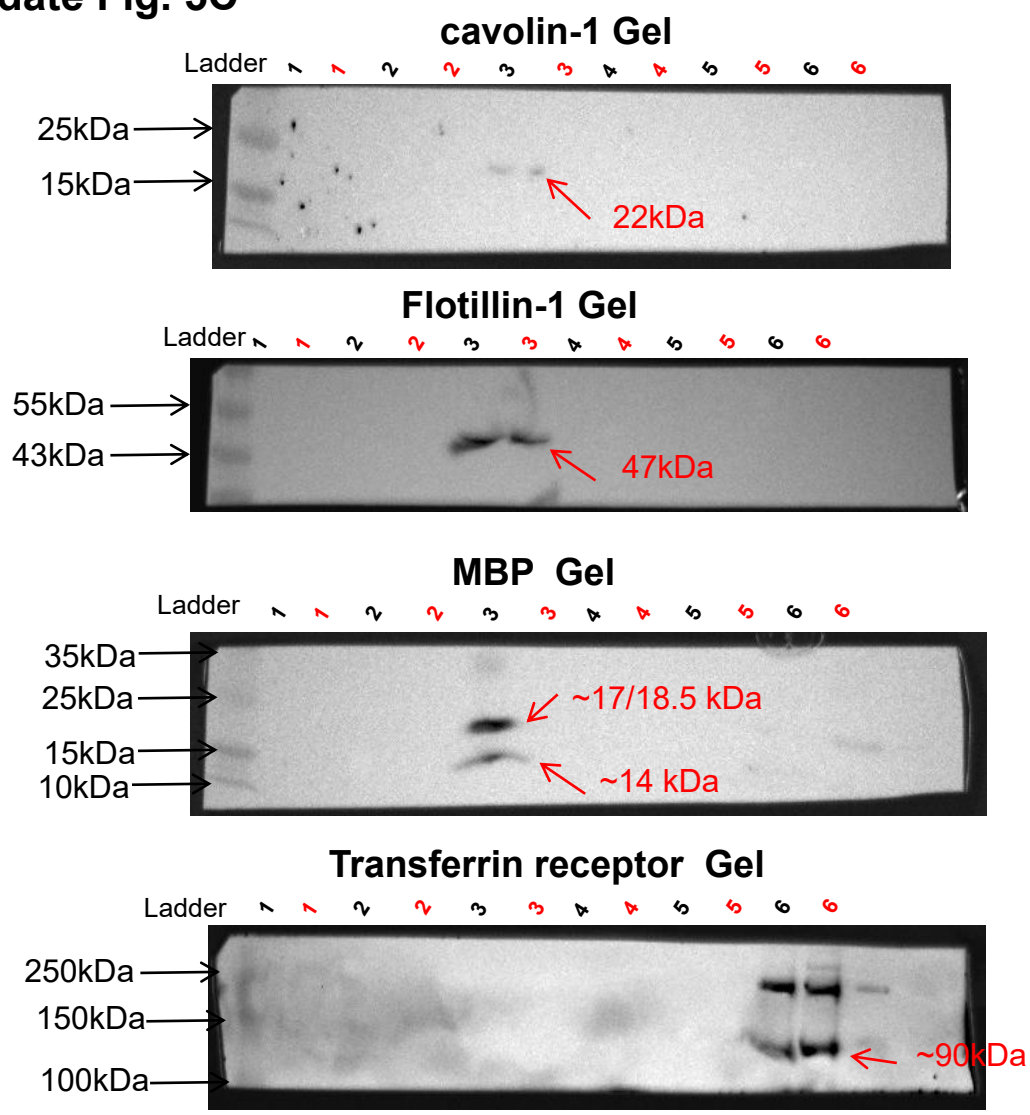

Supplementary Fig. 1B      CerS2 Gel

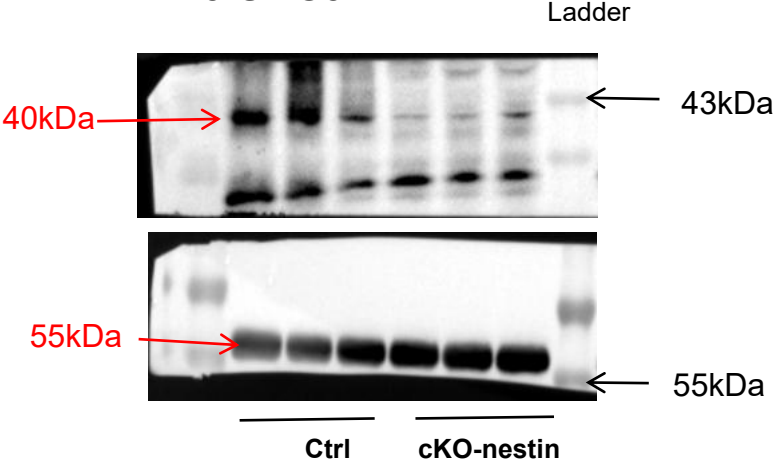

Supplementary Fig. 1A

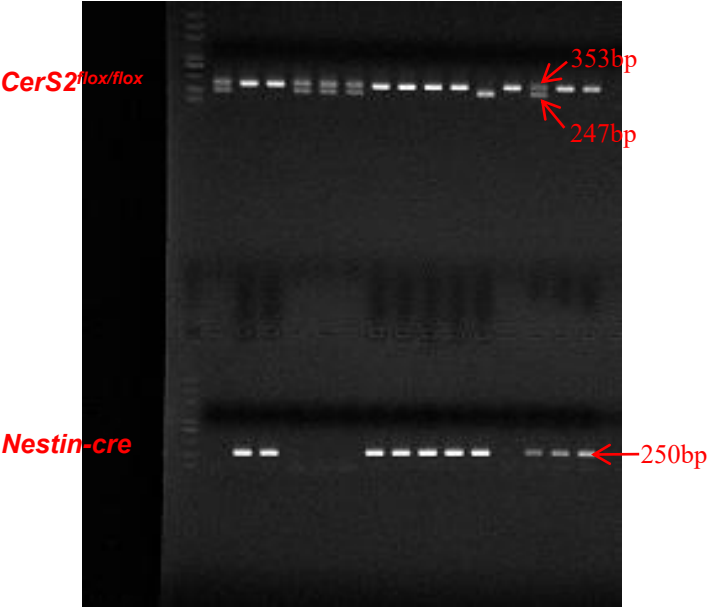

Supplement: Supplementary file 2 — Supplementary material 2 [file 12967_2026_7881_MOESM2_ESM.pdf]
